# Three New Dipeptide and Two New Polyketide Derivatives from the Mangrove-Derived Fungus *Talaromyces* sp.: Antioxidant Activity of Two Isolated Substances

**DOI:** 10.3390/md22120559

**Published:** 2024-12-14

**Authors:** Zhihao Zeng, Jian Cai, Yi Chen, Xinlong Li, Chunmei Chen, Yonghong Liu, Lalith Jayasinghe, Xuefeng Zhou

**Affiliations:** 1 CAS Key Laboratory of Tropical Marine Bio-resources and Ecology/Guangdong Key Laboratory of Marine Materia Medica, South China Sea Institute of Oceanology, Chinese Academy of Sciences, Guangzhou 510301, China; zengzhihao23@mails.ucas.ac.cn (Z.Z.); caijian@scsio.ac.cn (J.C.); chenyi221@mails.ucas.ac.cn (Y.C.); 13409379936@163.com (X.L.); chenchunmei18@mails.ucas.ac.cn (C.C.); yonghongliu@scsio.ac.cn (Y.L.); lalith.ja@nifs.ac.lk (L.J.); 2 University of Chinese Academy of Sciences, Beijing 100049, China; 3 Guangxi Key Laboratory of Marine Drugs, Institute of Marine Drugs, Guangxi University of Chinese Medicine, Nanning 530200, China; 4 National Institute of Fundamental Studies, Hantana Road, Kandy 200000, Sri Lanka

**Keywords:** dipeptide, polyketide, mangrove-derived fungus, *Talaromyces* sp., DPPH

## Abstract

Five new metabolites, including three cyclic dipeptide derivatives (**1**–**3**) and two new polyketides (**10**–**11**), together with nine known ones (**4**–**9** and **12**–**15**), were isolated from the mangrove-sediments-derived fungus *Talaromyces* sp. SCSIO 41431. Their structures were determined using detailed NMR, MS spectroscopic analyses, and quantum chemical calculations. X-ray single-crystal diffraction analysis of **1** was described. Compounds **13**–**15** demonstrated activity against *Staphylococcus aureus*, with MIC values ranging from 25 to 50 µg/mL. Compound **9** showed activity against *Escherichia coli*, *Streptococcus suis*, and *Erysipelothrix rhusiopathiae*, with an MIC value of 100 µg/mL. In addition, compounds **1** and **12** showed DPPH radical scavenging activity, with the EC_50_ of 27.62 and 29.34 µg/mL, compared to the positive control (ascorbic acid, EC_50_, 12.74 µg/mL).

## 1. Introduction

Mangroves, as highly productive ecosystems widely distributed along tropical and subtropical coasts, contain rich microbial resources [1,2]. A large number of structurally novel and biologically active natural products have been reported to be isolated from mangroves, making them one of the most important sources of marine natural products [3,4]. From January 2021 to May 2024, researchers isolated 165 novel secondary metabolites from 39 strains of fungi and 2 strains of actinomycetes from mangroves. Nearly half of these metabolites exhibited various biological activities, such as anti-inflammatory, antibacterial, cytotoxic, and antioxidant effects, thereby providing a substantial source of molecules for drug development [5].

*Talaromyces* sp. isolated from mangroves produce a wide range of biologically active secondary metabolites, mainly polyketides, alkaloids, and quinones [6,7,8,9]. Talaperoxides A-D produced by *Talaromyces flavus*, an endophytic fungus isolated from the mangrove plant *Sonneratia apetala*, were cytotoxic to five human cancer cells [10]. Talaromyone B produced by *Talaromyces stipitatus* SK-4, an endophytic fungus isolated from the mangrove plant *Acanthus ilicifolius*, showed anti-*bacillus subtilis* effects with a MIC value of 12.5 μg/mL [11].

**Figure 1:**
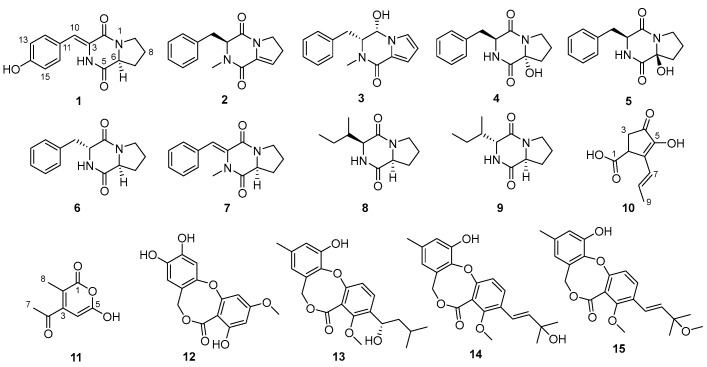
Structures of compounds **1**–**15**.

In our study, five new metabolites (**1**–**3** and **10**–**11**) (Figure 1) were isolated from the mangrove-sediment-derived fungus *Talaromyces* sp. SCSIO 41431. Herein, the specifics of the isolation, structural elucidation, and bioactive assessments of isolated compounds were reported.

## 2. Results and Discussion

### 2.1. Structural Determination

**Figure 2:**
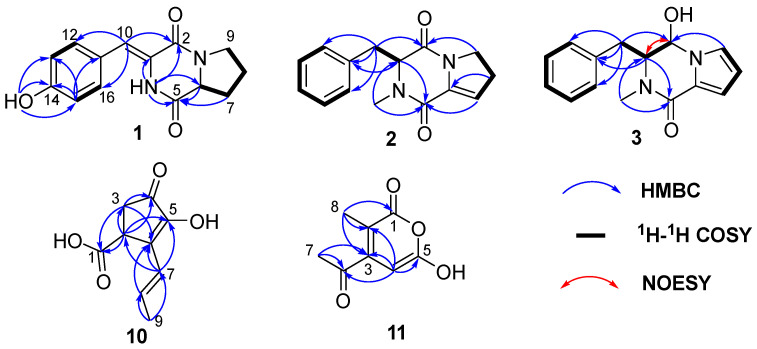
Key HMBC, ^1^H-^1^H COSY, and NOESY correlations of **1**–**3** and **10**–**11**.

**Figure 3:**
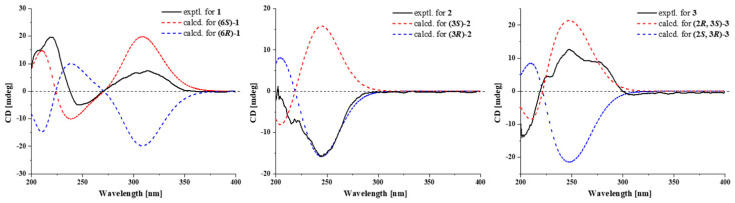
Experimental and calculated ECD spectra of compounds **1**–**3**.

**Figure 4:**
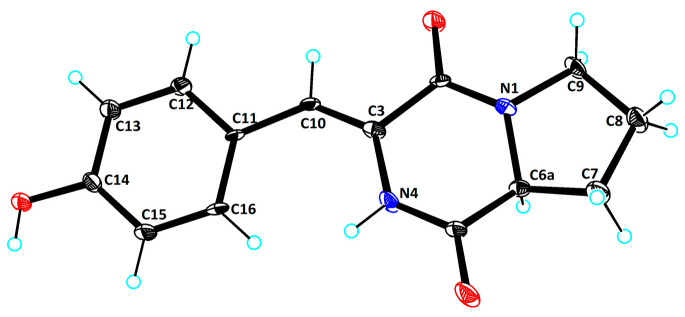
X-ray single-crystal diffraction of compound **1**.

Compound **1** was isolated as green needles, and its molecular formula of C_14_H_14_N_2_O_3_ was determined via HRESIMS data at *m*/*z* 259.1078 [M+H]+ (calculated for C_14_H_15_N_2_O_3_^+^, 259.1077). One-dimensional NMR (Table 1) and HSQC spectra of **1** showed the presence of five aromatic/olefinic protons [*δ*_C/H_ 115.5/6.59 (CH-10), 131.1/7.38 (CH-12), 115.5/6.78 (CH-13, 15), 131.41/7.38 (CH-16)], one methine [*δ*_C/H_ 58.2/4.34 (CH-6)], three methylenes [*δ*_C/H_ 28.0/2.22, 1.88 (CH_2_-7), 21.7/1.88 (CH_2_-8), 45.1/3.51 (CH_2_-9)], and five nonprotonated sp^2^ carbons [*δ*_C_ 159.2 (C-2), 124.3 (C-3), 167.1 (C-5), 126.1 (C-11), 157.6 (C-14)]. Additionally, the ^1^H NMR revealed the presence of two reactive hydrogens: NH (9.83, s) and 14-OH (9.76, s). The data above indicated that compound **1** possessed a skeleton resembling a cyclic dipeptide, composed of tyrosine and proline. Compounds **1** and **7** exhibited structural similarities, with the distinction that compound 7 is composed of phenylalanine and proline, while in compound 1, the C-3 and C-13 positions of tyrosine form a Δ^3,10^ double bond. This finding was further supported by the ^1^H-^1^H COSY and HMBC correlations (Figure 2). The ^1^H-^1^H COSY correlations of H-12/H-13, H-15/H-16, along with the HMBC correlations of H-15/C-14, C-13, C-11, and H-10/C-12, C-16, C-2, confirmed the presence of the tyrosine moiety with a Δ^3,10^ double bond. Additionally, the ^1^H-^1^H COSY correlations of H-6/H_2_-7/H_2_-8/H_2_-9, and the HMBC correlations of H_2_-7/C-5, H-6/C-5, and NH/C-2, C-3, C-5, C-6, provided evidence for the presence of proline. Furthermore, to determine the absolute configuration of the compound, ECD calculations were performed for the 6*S* and 6*R* configurations of compound **1**. The results showed that the experimental ECD curve fitted well with the calculated curve for 6*S* (Figure 3). Additionally, X-ray diffraction of **1** confirmed the accuracy of its planar structure and absolute configuration (Figure 4, CCDC 2401795). Consequently, compound **1** was identical to talarodipeptide A.

Compound **2** was isolated as a colourless oil, and the HRESIMS analysis determined its molecular formula to be C_15_H_16_N_2_O_2_, with an [M + H]^+^ value of *m*/*z* 257.1282 (calculated for C_15_H_17_N_2_O_2_^+^, 257.1285). The one-dimensional NMR (Table 1) showed that its structure was similar to that of compound **7**, classifying it as a derivative of a cyclic dipeptide. Unlike compound **7**(Δ^3,10^ double bond) the position of the double bond in compound **2** (Δ^6,7^ double bond) was different. Compared to compound **1**, compound **2** lacked the OH group at C-14 and the Δ^3,10^ double bond, as confirmed by the ^1^H-^1^H COSY correlations of H-12/H-13/H-14/H-15/H-16 and H-3/H_2_-10 (Figure 2). Additionally, compound **2** contained an N-CH_3_ group and a Δ^6,7^ double bond, as revealed by the HMBC correlations of H_3_-17/C-3, 5 and H_2_-8/C-6. By comparing calculated ECD with experimental ECD (Figure 3), the absolute configuration was determined to be 3*R*. Compound 2 is described here as a new natural product, designated talarodipeptide B.

Compound **3** was isolated as a brown oil, and its molecular formula was determined to be C_15_H_16_N_2_O_2_ using the HRESIMS, yielding an [M+H]^+^ value of *m*/*z* 257.1291 (calculated for C_15_H_17_N_2_O_2_^+^, 257.1285), with a total of 9 degrees of unsaturation. One-dimensional NMR (Table 1) indicated that its structure was similar to that of compound **2**, classifying it as a derivative of a cyclic dipeptide. In contrast to compound **2**, the C=O at C-2 in compound **3** had been reduced to an OH group and formed a Δ^8, 9^ double bond. This deduction was supported by the ^1^H-^1^H COSY correlations of H_2_-10/H-1/H-2 and H-7/H-8/H-9. The NOESY correlations between H-2 and H-3, along with the absence of NOESY signals between H-2 and H_2_-10, suggested that the relative configuration of compound **3** was 2R*, 3S*. Comparing calculated ECD (for 2*R*, 3*S* and 2*S*, 3*R*) with experimental ECD data, the absolute configuration of compound **3** was determined to be 2*R*, 3*S* (Figure 3), and it had been named talarodipeptide C.

Compound **10** was obtained as brown oil and had its molecular formula determined as C_9_H_10_O_4_ by HRESIMS *m*/*z* 181.0498 [M-H]^−^ (calculated for C_9_H_9_O_4_^−^, 181.0506). The one-dimensional NMR (Table 2) and HSQC spectra indicated the presence of two olefinic protons (*δ*_C/H_ 123.8/6.49 and 131.1/6.15), one methine (*δ*_C/H_ 40.1/3.64), one methylene (*δ*_C/H_ 36.4/2.62, 2.28), one methyl (*δ*_C/H_ 19.0/1.84), one carboxyl group (*δ*_C_ 175.0), one carbonyl group (*δ*_C_ 200.2), and two nonprotonated *sp*^2^ carbons (δ_C_ 149.3 and 137.5). The HMBC correlations of H-2/C-1, 3, 4, 5 and H-3/C-1, 4, 6 (Figure 2) confirmed that the carboxyl group was attached to the five-membered ring at C-2, 3, 4, 5, and 6. The HMBC correlations of H-9/C-7, 8, H-8/C-6, and H-7/C-6, 5, 2 indicated the presence of a butene fragment at C-6. The coupling constant between H-7 and H-8 (15.9 Hz) suggested that the double bond was in the *E* configuration. Additionally, its optical rotation ([*α*${]}_{D}^{25}$ −0.07) and ECD curve (Supporting Information) were close to zero, indicating the presence of a pair of enantiomers. The planar structure of compound **10** had been elucidated and named talaropolyketone A.

Compound **11** was obtained as brown solid and had its molecular formula determined as C_8_H_8_O_4_ by HRESIMS *m*/*z* 169.0494 [M+H]+ (calculated for C_8_H_9_O_4_^+^, 169.0495), indicating 5 degrees of unsaturation. One-dimensional NMR (Table 2) and HSQC spectra showed one olefinic proton (δ_C/H_ 104.8/6.85), two methyl groups (δ_C/H_ 25.6/2.14, 9.2/1.84), one ester carbonyl (δ_C_ 163.2), one ketone carbonyl (δ_C_ 191.1), and three nonprotonated olefinic carbons (δ_C_ 104.0, 151.7, 104.8, 164.2). The HMBC correlations of H-7/C-6, 3 indicate an acetyl group is attached at C-3. These data confirmed that compound **11** was a lactone within a six-membered ring. The HMBC correlations of H-8/C-1, 2, 3 showed that CH_3_-8 was connected to C-2. Additionally, the HMBC correlations of H-4/C-2, 3, 5, 6 confirmed that the olefinic proton is connected to C-3 and C-5. Therefore, the structure of compound **11** was determined and named talaropolyketone B.

Meanwhile, the other ten known compounds were identified as cyclo(D-6-Hyp-L-Phe) (**4**) [12], cyclo(l-6-Hyp-L-Phe) (**5**) [12], cyclo-(Pro-Phe) (**6**) [13], (*Z*)-3-benzylidene-2-methylhexahydropyrrolo [1,2] pyrazine-1,4-dione (**7**) [14], cyclo-(L-Ile-L-Pro) (**8**) [15], cyclo(D)-Pro-(D)-Leu (**9**) [15], alterlactone (**12**) [16], penicillide (**13**) [17], dehydroisopenicillide (**14**) [18], and 3′-O-Methyldehydroisopenicillide (**15**) [18], respectively, by comparing their NMR data (Supporting Information) to previous reports.

### 2.2. Bioactivity Assay

**Figure 5:**
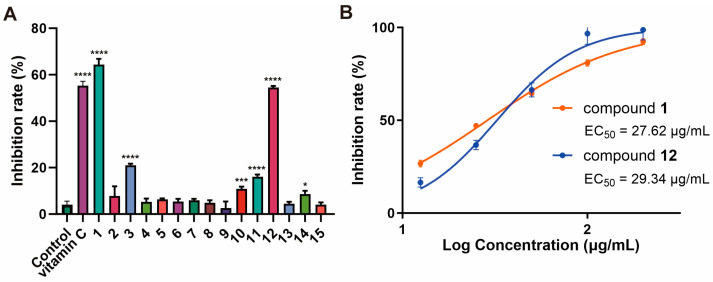
(**A**) DPPH radical scavenging activity of the compounds **1**–**15** (50 µg/mL). All experiments were performed at least three times. The data are presented as the mean ± SD of representative experiments. Statistical significance was determined with one-way ANOVA. * *p* < 0.05, *** *p* < 0.001, and **** *p* < 0.0001 were considered statistically significant. (**B**) Compounds **1** and **12** of DPPH scavenging activity of EC_50_.

Compounds **1**–**15** were evaluated for their antibacterial (*Escherichia coli, Staphylococcus aureus, Streptococcus suis, Erysipelothrix rhusiopathiae, Micrococcus luteus), antifungal (Colletotrichum acutatum, Curvularia australiensis, Fusarium oxysporum*), phosphodiesterase 4 (PDE4) inhibitory, NF-κB inhibitory, and antioxidant activities. Compounds **13**–**15** exhibited weak activities against Staphylococcus aureus, with the MIC values of 50, 50, and 25 µg/mL. Compound **9** exhibited activities against *Escherichia coli, Streptococcus suis, and Erysipelothrix rhusiopathiae*, with MIC values of 100 µg/mL. The obtained compounds showed no activity against fungi, PDE4, and NF-κB. In addition, compounds **1**, **3**, and **10**–**12** at 50 μg/mL (vitamin C, 12.5 μg/mL) exhibited significant antioxidant activity, with compounds **1** and **12** showing DPPH radical scavenging rates of EC_50_ values of 27.62 and 29.34 µg/mL, respectively, compared to the positive control (vitamin C, with an EC_50_ of 12.74 µg/mL) (Figure 5).

Natural antioxidants can scavenge free radicals, and the development of novel antioxidants has significant implications for health and disease prevention [19]. This study found that dipeptides and polymer derivatives possess antioxidant activity, particularly emphasizing that the novel cyclic dipeptide compounds are worthy of further exploration. The structure–activity relationship (SAR) analysis revealed that the number and position of hydroxyl groups and double bonds significantly influence the intensity of antioxidant activity. Compared to compound **7**, the presence of the 14-OH group and the Δ^3,10^ double bond in compound **1** significantly enhanced its antioxidant activity. Similarly, the hydroxyl group and Δ^3,10^ double bond in compound **3** increased its activity compared to compound **2**. Likewise, compounds **10**, **11**, and **12** exhibit enhanced activity due to differences in the positions of their hydroxyl groups. Cyclic dipeptide compound **1** demonstrated the strongest activity, indicating that the tyrosine residue was an important moiety for radical scavenging activity. The presence of the tyrosine residue, which had electron/hydrogen donating capabilities, was a driving force for the dipeptide’s ability to scavenge free radicals. Additionally, adjacent residues were influenced by spatial effects, hydrophobicity, and hydrogen bonding, all of which could affect their activity [20]. Therefore, the novel cyclic dipeptides hold promise for development as new antioxidants.

## 3. Materials and Methods

### 3.1. General Experimental Procedures

Optical rotations were measured using an Anton Paar MPC500 polarimeter (Anton, Graz, Austria). UV spectra were recorded on a Shimadzu UV-2600 PC spectrometer (Shimadzu, Beijing, China), while IR spectra were determined with an IR Affinity-1 spectrometer (Shimadzu). ECD spectra were obtained using a Chirascan circular dichroism spectrometer (Applied Photophysics, Leatherhead Surrey, UK). High-resolution electrospray ionization mass spectrometry (HRESIMS) was performed on a Bruker maXis Q-TOF mass spectrometer (Bruker BioSpin International AG, Fällanden, Switzerland). NMR spectra were collected on a Quantum-I Plus 500 MHz spectrometer (Q-one Instrument Co., LTD, Wuhan, China), operating at 500 MHz for ^1^H NMR and 125 MHz for ^13^C NMR, with tetramethylsilane as the internal standard. Semipreparative high-performance liquid chromatography (HPLC) was conducted on a Hitachi Primaide with a DAD detector, using ODS columns (ChromCore 120 C18, 10 × 250 mm, 5 µm; YMC-pack ODS-A, 10 × 250 mm, 5 µm).

### 3.2. Fungal Material

The fungal strain SCSIO 41431 was isolated from the sediment sample of the mangrove in Gaoqiao Mangrove, Zhanjiang. It was stored in the CAS Key Laboratory of Tropical Marine Bioresources and Ecology, South China Sea Institute of Oceanology, Chinese Academy of Sciences, Guangzhou, China. The strain was designated *Talaromyces* sp. SCSIO 41431 was based on BLAST analysis of the ITS sequence (Supporting Information). Finally, the sequence was deposited in GenBank with the accession number PQ590239.

### 3.3. Fermentation and Extraction

The fungal strain *Talaromyces* sp. SCSIO 41431 was statically cultivated in potato dextrose broth (PDB) medium, followed by culture in 200 mL seed medium (1.5% malt extract, 2.0% sea salt) in 1 L Erlenmeyer flasks at 28 °C for 3 days on a rotary shaker at 180 rpm. A large-scale fermentation was then conducted at 26 °C for 28 days using a rice medium (200 g rice, 1% tryptone, 1.5% malt extract, 2% sea salt, 230 mL H_2_O) in 1 L flasks (60 flasks total) under static conditions. The entire fermented culture was extracted with ethyl acetate three times, yielding a brown extract weighing 180.5 g.

### 3.4. Isolation and Purification

The whole ethyl acetate extract was subjected to a silica gel vacuum liquid chromatography using a step gradient elution of petroleum ether (PE)-dichloromethane (DCM) (*ν:ν* 1:0, 1:1, 0:1), DCM-CH_3_OH (*ν:ν* 99:1, 97:3, 95:5, 90:10, 80:20, 50:50, 0:100), to yield 15 fractions (Frs. 1–15) in the light of TLC profiles. Fr. 8 was divided into 15 subfractions (Frs. 8-1–Fr. 8-15) by ODS silica gel eluting with CH_3_OH/H_2_O (5–100%). Fr. 8-2 was separated by semipreparative HPLC (38% CH_3_OH/H_2_O, 2.0 mL/min) to eight subfractions (Fr. 8-2-1–Fr. 8-2-8). Compounds **8** (16.4 mg, *t*_R_ 13.0 min) and **9** (17.7 mg, *t*_R_ 14.9 min) were further purified from Fr. 8-2-4 by semipreparative HPLC (18% CH_3_CN/H_2_O, 3.0 mL/min). Fr. 4-2-6 was separated by semipreparative HPLC (18% CH_3_CN/H_2_O, 3.0 mL/min) to gain compounds **5** (2.1 mg, *t*_R_ 14.0 min), **4** (2.0 mg, *t*_R_ 17.0 min), and **6** (67.4 mg, *t*_R_ 19.4 min). Compound **2** (1.3 mg, *t*_R_ 7.5 min) was further obtained from Fr. 8-7 by semipreparative HPLC (55% CH_3_OH/H_2_O, 3 mL/min). Compound **7** (25.0 mg, *t*_R_ 7.0 min) was obtained from Fr. 8-8 by semipreparative HPLC (50% CH_3_CN /H_2_O, 3.0 mL/min). Compound **14** (6.3 mg, *t*_R_ 19.5 min) and **15** (5.9 mg, *t*_R_ 23.1 min) was obtained from Fr. 8-9 by semipreparative HPLC (75% CH_3_OH /H_2_O, 3.0 mL/min). Fr. 11 was divided into 16 subfractions (Frs. 11-1–Fr. 11-16) by ODS silica gel eluting with CH_3_OH/H_2_O (5–100%). Compound **11** (12.1 mg, *t*_R_ 9.5 min) was separated from Fr. 11-5 by semipreparative HPLC (27% CH_3_CN/H_2_O, 0.04% formic acid, 3.0 mL/min). Compound **12** (8.2 mg, *t*_R_ 20.2 min) and compound **3** (4.5 mg, *t*_R_ 23.5 min) were further purified from Fr. 11-10 by semipreparative HPLC (61% CH_3_OH/H_2_O, 0.04% formic acid, 3.0 mL/min). Compound **13** (161.6 mg, *t*_R_ 25.2 min) was further purified from Fr. 11-14 by semipreparative HPLC (61% CH_3_OH/H_2_O, 0.04% formic acid, 3.0 mL/min). Fr. 12 was divided into 15 subfractions (Frs. 12-1–Fr. 12-15) by ODS silica gel eluting with CH_3_OH/H_2_O (5–100%). Fr. 12-4 was separated by semipreparative HPLC (22% CH_3_CN/H_2_O, 3.0 mL/min) to gain compound **1** (11.3 mg, *t*_R_ 10.6 min). Fr. 13-3 was separated by semipreparative HPLC (40% CH_3_CN/H_2_O, 3.0 mL/min) to gain compound **10** (3.0 mg, *t*_R_ 7.2 min).

### 3.5. Spectroscopic Data of Compounds

Talaropolyketone A (**1**): green needles; [*α*${]}_{D}^{25}$ +9.3 (*c* 0.1, CH_3_OH); UV (CH_3_OH) *λ*_max_ (log*ε*) 224 (3.81), 316 (3.88) nm; ECD (0.77 mM, CH_3_OH) *λ*_max_ (Δ*ε*) 221 (+7.69), 246 (–1.95), 313 (+2.92); IR (film) *ν*_max_ 3362, 2959, 2922, 1094, 1026, 677 cm^−1^; ^1^H and ^13^C NMR data, see Table 1; HRESIMS *m*/*z* 259.1078 [M+H]+ (calculated for C_14_H_15_N_2_O_3_^+^, 259.1077).

Talaropolyketone B (**2**): colourless oil; [*α*${]}_{D}^{25}$ –6.9 (*c* 0.1, CH_3_OH); UV (CH_3_OH) *λ*_max_ (log*ε*) 200 (4.27), 242 (4.03) nm; ECD (0.78 mM, CH_3_OH) *λ*_max_ (Δ*ε*) 245 (–6.13); IR (film) *ν*_max_ 3387, 2963, 2943, 1682, 1634, 1402, 1045, 702 cm^−1^; ^1^H and ^13^C NMR data, see Table 1; HRESIMS *m*/*z* 257.1282 [M+H]+ (calculated for C_15_H_17_N_2_O_2_^+^, 257.1285).

Talaropolyketone C (**3**): brown oil; [*α*${]}_{D}^{25}$ +6.1 (*c* 0.1, CH_3_OH); UV (CH_3_OH) *λ*_max_ (log*ε*) 200 (4.30), 274 (3.86) nm; ECD (0.78 mM, CH_3_OH) *λ*_max_ (Δ*ε*) 201 (–5.45), 248 (+4.93); IR (film) *ν*_max_ 3296, 2932, 1614, 1549, 1456, 1126, 745 cm^−1^; ^1^H and ^13^C NMR data, see Table 1; HRESIMS *m*/*z* 257.1291 [M+H]+ (calculated for C_15_H_17_N_2_O_2_^+^, 253.1285).

Talaropolyketone D (**10**): brown oil; [*α*${]}_{D}^{25}$ -0.07 (*c* 0.1, CH_3_OH); UV (CH_3_OH) *λ*_max_ (log*ε*) 200 (3.72), 298 (3.63) nm; IR (film) *ν*_max_ 3437, 2967, 2932, 1697, 1186, 1136, 974, 721 cm^−1^; ^1^H and ^13^C NMR data, see Table 2; HRESIMS *m*/*z* 181.0498 [M-H]^−^ (calculated for C_9_H_9_O_4_^−^, 181.0506).

Talaropolyketone E (**11**): brown solid; UV (CH_3_OH) *λ*_max_ (log*ε*) 202 (3.80), 226 (4.05), 328 (3.59) nm; IR (film) *ν*_max_ 3354, 1682, 1645, 1406, 1219, 1024, 750 cm^−1^; ^1^H and ^13^C NMR data, see Table 2; HRESIMS *m*/*z* 169.0494 [M+H]+ (calculated for C_8_H_9_O_4_^+^, 169.0495).

### 3.6. X-Ray Crystallographic Analysis

The crystallographic data for compound **1**, obtained through slow evaporation in methanol, were collected using an XtaLAB PRO single-crystal diffractometer with Cu K*α* radiation. The X-ray crystal structures were solved using SHELXS97, expanded via difference Fourier techniques, and refined through full-matrix least-squares calculations. Non-hydrogen atoms were refined anisotropically, while hydrogen atoms were fixed at calculated positions. The crystallographic data for these compounds have been deposited in the Cambridge Crystallographic Data Centre.

Crystal data for **1**: C_14_H_14_N_2_O_3_ (M = 258.27 g/mol): monoclinic, space group P2_1_/n (no. 14), a = 5.8882 (2) Å, b = 11.5552 (3) Å, c = 17.5786 (3) Å, β = 94.906 (2)°, V = 1191.65 (5) Å^3^,Z = 4, T = 99.97 (17) K, μ(Cu Kα) = 0.848 mm^−1^, D_calc_ = 1.440 g/cm^3^, 5047 reflections measured (9.168° ≤ 2Θ ≤ 133.186°), 2084 unique (R_int_ = 0.0460, R_sigma_ = 0.0528) which were used in all calculations. The final R_1_ was 0.1370 (I > 2σ(I)) and wR_2_ was 0.3178 (all data) (Figure 4, CCDC 2401795).

### 3.7. ECD Computation Section

Compounds **1**–**3** were analyzed using Spartan’14, employing the MMFF molecular force field to conduct a conformational search for potential isomers. The most stable conformers, representing the top 5%, were then optimized in methanol solvent at the B3LYP/6-31G (d) level using Gaussian 09 (D.01, Pittsburgh, PA, USA). A TDDFT polarizable continuum model at the B3LYP/6-311G (d, p) level was utilized to calculate the optimized low-energy conformations. [21]. The calculated ECD spectra were generated using GaussView (6.0.16, Pittsburgh, PA, USA) with a half-bandwidth of 0.3 eV and weighted by Boltzmann distribution. Both the calculated ECD and experimental ECD spectra were plotted using Origin 2021.

### 3.8. Antioxidant Activity Assay

The obtained compounds were evaluated for its antioxidant activities against DPPH. The effect of the compounds on DPPH radical were estimated, as previously reported [22]. In summary, a methanolic DPPH solution was supplemented with compounds to achieve final concentrations of 12.5–200 μg/mL. The mixture was shaken and allowed to stand for 30 min at room temperature in the dark, after which the OD_517_ values were measured using a PerkinElmer Enspire Multi-mode detector (Thermo Scientific, Bremen, Germany). Vitamin C served as the positive control. The free radical scavenging rate (K%) was calculated from the OD_517_ values, and EC_50_ was determined using Origin 2021.

### 3.9. Antimicrobial Activity Assay

The antimicrobial activities against four bacteria *(Escherichia coli, Staphylococcus aureus, Streptococcus suis, Erysipelothrix rhusiopathiae, Micrococcus luteus*) and three fungi (*Colletotrichum acutatum, Curvularia australiensis, Fusarium oxysporum*) were evaluated in 96-well plates with a twofold serial dilution method described previously [23]. After 48 h of incubation, the optical density of the medium was measured at 600 nm (OD600) using a PerkinElmer Enspire Multi-mode detector. Incubation conditions were as follows: fungi PDB medium (Potato extract 0.4%, Glucose 2%), 26 °C, 48 h; Bacteria LB medium (Yeast extract 0.5%, Tryptone 1%, NaCl 0.5%), 26 °C, 48 h.

### 3.10. PDE4 and NF-κB Inhibitory Screening Assays

PDE4 inhibition screening test procedures, as previously reported using the phosphodiesterase scintillation proximity assay [22,24]. To assess the enzymatic activity of the catalytic domains, we used 3H-cAMP or 3H-cGMP as substrates. The experiments were performed in assay buffer containing Tris-HCl, MgCl_2_ or MnCl_2_, DTT, and 3H-cAMP or 3H-cGMP, and the protein concentration was set at 2 nM. The reaction was terminated after 15 min of incubation, and the products were separated by precipitation, while unreacted substrates retained in the supernatant. The radioactivity of the supernatant was measured using a liquid scintillation counter.

NF-κB inhibition screening test procedures, as previously reported using luciferase reporter gene assay [25]. RAW264.7 cells transfected with a luciferase reporter gene were cultured in 96-well plates and pretreated with test compounds (20 μM) and BAY11-7082 (NF-κB inhibitor, 5 μM, purchased from Sigma-Aldrich as a positive control) for 30 min, followed by stimulation of the cells with 5 μg/mL LPS for 8 h. At the end of the experiment, the cells were collected, and luciferase activity was measured using a luciferase assay system (Promega, Madison, WI, USA), and each sample was repeated three times.

## 4. Conclusions

In conclusion, five new metabolites (**1**–**3** and **10**–**11**), together with ten known ones (**4**–**9** and **12**–**15**), were isolated from the mangrove-sediments-derived fungus *Talaromyces* sp. SCSIO 41431. Their structures were determined by extensive spectroscopic analyses, ECD calculations, and X-ray single-crystal diffraction. Compounds **9** and **13**–**15** exhibited weak activities against pathogenic bacteria. The obtained compounds showed no activity against PDE4 and NF-κB enzymes. Moreover, compounds **1** and **13** showed DPPH radical scavenging activity, with the EC_50_ of 27.62 and 29.34 µg/mL.

## Figures and Tables

**Table 1:** ^1^H (500 MHz) and ^13^C (125 MHz) NMR data of **1**–**3** in DMSO-*d*_6_.

Pos.	1		2		3	
	*δ*_C_ Type	*δ*_H_ (*J* in Hz)	*δ*_C_ Type	*δ*_H_ (*J* in Hz)	*δ*_C_ Type	*δ*_H_ (*J* in Hz)
2	159.2, C		77.0, CH	5.68, d (3.7)	165.5, C	
3	124.3, C		63.7, CH	3.92, dt (9.0, 3.7)	62.8, CH	4.37, t (4.4)
5	167.1, C		158.1, C		158.8, C	
6	58.2, CH	4.34, m	122.9, C		127.0, C	
7	28.0, CH_2_	2.20, m; 1.88, overlapped	120.3, CH	6.60, dd (3.7, 1.6)	114.4, CH	5.40, t (7.7)
8	21.7, CH_2_	1.88, overlapped	108.9, CH	6.15, dd (3.7, 2.7)	28.6, CH_2_	2.04, m
9	45.1, CH_2_	3.51, m	112.2, CH	6.99, dd (2.7, 1.6)	59.8, CH_2_	3.23, m
10	115.5, CH	6.59, s	33.3, CH_2_	2.56, m; 3.08, dd (13.6, 5.2)	30.7, CH_2_	3.04, dd (13.8, 3.8); 3.15, dd (13.8, 5.1)
11	126.1, C		138.0, C		134.8, C	
12	131.1, CH	7.38, d (8.3)	129.4, CH	7.16, d (6.8)	129.7, CH	7.03, m
13	115.5, CH	6.78, d (8.4)	128.3, CH	7.28, d (7.4)	128.1, CH	7.21, overlapped
14	157.6, C		126.3, CH	7.21, m	126.9, CH	7.21, overlapped
15	115.5, CH	6.78, d (8.4)	128.3, CH	7.28, d (7.4)	128.1, CH	7.21, overlapped
16	131.1, CH	7.38, d (8.3)	129.4, CH	7.16, d (6.8)	129.7, CH	7.03, m
17			32.9, CH_3_	2.54, s	32.3, CH_3_	2.97, s
NH		9.83, s			NH	9.79, s
14-OH		9.76, s				

**Table 2:** ^1^H (500 MHz) and ^13^C (125 MHz) NMR data of **10**–**11** in DMSO-*d*_6_.

Pos.	10		11	
	*δ*_C_ Type	*δ*_H_ (*J* in Hz)	*δ*_C_ Type	*δ*_H_ (*J* in Hz)
1	175.0, C		163.2, C	
2	40.1, CH	3.64, d (7.0)	104.0, C	
3	36.4, CH_2_	2.62, dd (18.4, 7.0); 2.28, d (18.4)	151.7, C	
4	200.2, C		104.8, CH	6.85, s
5	149.3, C		164.2, C	
6	137.5, C		191.1, C	
7	123.8, CH	6.49, d (15.9)	25.6, CH_3_	2.14, s
8	131.2, CH	6.15, dq (15.9, 6.8)	9.2, CH_3_	1.84, s
9	19.0, CH_3_	1.84, d (6.8)		
5-OH		9.78, s		

## Data Availability

Data are contained within the article and Supplementary Materials.

## Supplementary Materials

The following supporting information can be downloaded at https://www.mdpi.com/article/10.3390/md22120559/s1: Figures S1–S43: The NMR, HRESIMS, UV, and IR spectra of **1**–**3** and **10**–**11**; physicochemical data of **4**–**9** and **12**–**15**; ITS sequence data of the strain.

## References

[1] Barbier E.B., Koch E.W., Silliman B.R., Hacker S.D., Wolanski E., Primavera J., Granek E.F., Polasky S., Aswani S., Cramer L.A. (2008). Coastal Ecosystem-Based Management with Nonlinear Ecological Functions and Values. Science.

[2] Thatoi H., Behera B.C., Mishra R.R., Dutta S.K. (2013). Biodiversity and Biotechnological Potential of Microorganisms from Mangrove Ecosystems: A Review. Ann. Microbiol..

[3] Xu J. (2015). Bioactive Natural Products Derived from Mangrove-Associated Microbes. RSC Adv..

[4] Li K., Chen S., Pang X., Cai J., Zhang X., Liu Y., Zhu Y., Zhou X. (2022). Natural Products from Mangrove Sediments-Derived Microbes: Structural Diversity, Bioactivities, Biosynthesis, and Total Synthesis. Eur. J. Med. Chem..

[5] Yu Y., Wang Z., Xiong D., Zhou L., Kong F., Wang Q. (2024). New Secondary Metabolites of Mangrove-Associated Strains. Mar. Drugs.

[6] Liu F., Cai X.-L., Yang H., Xia X.-K., Guo Z.-Y., Yuan J., Li M.-F., She Z.-G., Lin Y.-C. (2010). The Bioactive Metabolites of the Mangrove Endophytic Fungus *Talaromyces* sp. ZH-154 Isolated from *Kandelia candel* (L.) Druce. Planta Med..

[7] Liu H., Chen S., Liu W., Liu Y., Huang X., She Z. (2016). Polyketides with Immunosuppressive Activities from Mangrove Endophytic Fungus *Penicillium* sp. ZJ-SY2. Mar. Drugs.

[8] Li J., Chen C., Fang T., Wu L., Liu W., Tang J., Long Y. (2022). New Steroid and Isocoumarin from the Mangrove Endophytic Fungus *Talaromyces* sp. SCNU-F0041. Molecules.

[9] Nicoletti R., Salvatore M., Andolfi A. (2018). Secondary Metabolites of Mangrove-Associated Strains of *Talaromyces*. Mar. Drugs.

[10] Li H., Huang H., Shao C., Huang H., Jiang J., Zhu X., Liu Y., Liu L., Lu Y., Li M. (2011). Cytotoxic Norsesquiterpene Peroxides from the Endophytic Fungus *Talaromyces flavus* Isolated from the Mangrove Plant *Sonneratia apetala*. J. Nat. Prod..

[11] Cai R., Chen S., Long Y., Li C., Huang X., She Z. (2017). Depsidones from *Talaromyces stipitatus* SK-4, an Endophytic Fungus of the Mangrove Plant Acanthus Ilicifolius. Phytochem. Lett..

[12] Park Y.C., Gunasekera S.P., Lopez J.V., McCarthy P.J., Wright A.E. (2006). Metabolites from the Marine-Derived Fungus *Chromocleista* sp. Isolated from a Deep-Water Sediment Sample Collected in the Gulf of Mexico. J. Nat. Prod..

[13] Sun Y., Wang C., Du G., Deng W., Yang H., Li R., Xu Q., Guo Q. (2022). Two Nematicidal Compounds from *Lysinimonas* M4 against the Pine Wood Nematode, *Bursaphelenchus xylophilus*. Forests.

[14] Lv M., Tan M., Lu L., Bao S., Guo Z., Deng Z., Zhou K. (2018). Secondary Metabolites from Endophytes of Elaeocarpus Decipiens Hemsl. with Co-Cultivation Method. J. China Three Gorges Univ. Sci..

[15] Guo Z.K., Wang R., Chen F.X., Liu T.M., Yang M.Q. (2018). Bioactive Aromatic Metabolites from the Sea Urchin-Derived Actinomycete *Streptomyces spectabilis* Strain HDa1. Phytochem. Lett..

[16] Aly A.H., Edrada-Ebel R., Indriani I.D., Wray V., Müller W.E.G., Totzke F., Zirrgiebel U., Schächtele C., Kubbutat M.H.G., Lin W.H. (2008). Cytotoxic Metabolites from the Fungal Endophyte *Alternaria* sp. and Their Subsequent Detection in Its Host Plant *Polygonum senegalense*. J. Nat. Prod..

[17] Jeon H., Shim S.H. (2020). Chemical Constituents of the Endophyte *Penicillium* sp. Isolated from *Artemisia princeps*. Chem. Nat. Compd..

[18] Xia X., Kim S., Liu C., Shim S. (2016). Secondary Metabolites Produced by an Endophytic Fungus *Pestalotiopsis sydowiana* and Their 20S Proteasome Inhibitory Activities. Molecules.

[19] Gulcin İ. (2020). Antioxidants and Antioxidant Methods: An Updated Overview. Arch. Toxicol..

[20] Zheng L., Zhao Y., Dong H., Su G., Zhao M. (2016). Structure-Activity Relationship of Antioxidant Dipeptides: Dominant Role of Tyr, Trp, Cys and Met Residues. J. Funct. Foods.

[21] Luo X., Lin X., Tao H., Wang J., Li J., Yang B., Zhou X., Liu Y. (2018). Isochromophilones A-F, Cytotoxic Chloroazaphilones from the Marine Mangrove Endophytic Fungus *Diaporthe* sp. SCSIO 41011. J. Nat. Prod..

[22] Chen Y., Cai J., Xia Z., Chen C., Liu Y., Jayasinghe L., Wang X., Zhou X. (2024). New Bioactive Polyketides from the Mangrove-Derived Fungus *Penicillium* sp. SCSIO 41411. Mar. Drugs.

[23] Cai J., Wang X., Yang Z., Tan Y., Peng B., Liu Y., Zhou X. (2022). Thiodiketopiperazines and Alkane Derivatives Produced by the Mangrove Sediment-Derived Fungus *Penicillium ludwigii* SCSIO 41408. Front. Microbiol..

[24] Cai J., Zhou Q., Qi X., Zhang F., Yang J., Chen C., Zhang K., Chen Z., Luo H.-B., Liu Y. (2024). Discovery of Oxidized P-Terphenyls as Phosphodiesterase 4 Inhibitors from Marine-Derived Fungi. J. Nat. Prod..

[25] Cai J., Gao L., Wang Y., Zheng Y., Lin X., Zhou P., Chen C., Liu K., Tang L., Liu Y. (2024). Discovery of a Novel Anti-Osteoporotic Agent from Marine Fungus-Derived Structurally Diverse Sirenins. Eur. J. Med. Chem..

