# Supplementary material for: Three New Dipeptide and Two New Polyketide Derivatives from the Mangrove-Derived Fungus Talaromyces sp.: Antioxidant Activity of Two Isolated Substances
Source: Mar Drugs. 2024 Dec 14;22(12):559. doi: 10.3390/md22120559 (PMC11677792; doi:10.3390/md22120559)
Supplement: Supplementary file 1 [file marinedrugs-22-00559-s001.zip › marinedrugs-3364752-supplementary.pdf]

Supporting Information

Three New Dipeptide and Two New Polyketide Derivatives from the Mangrove-Derived Fungus *Talaromyces* sp.: Antioxidant Activity of Two Isolated Substances

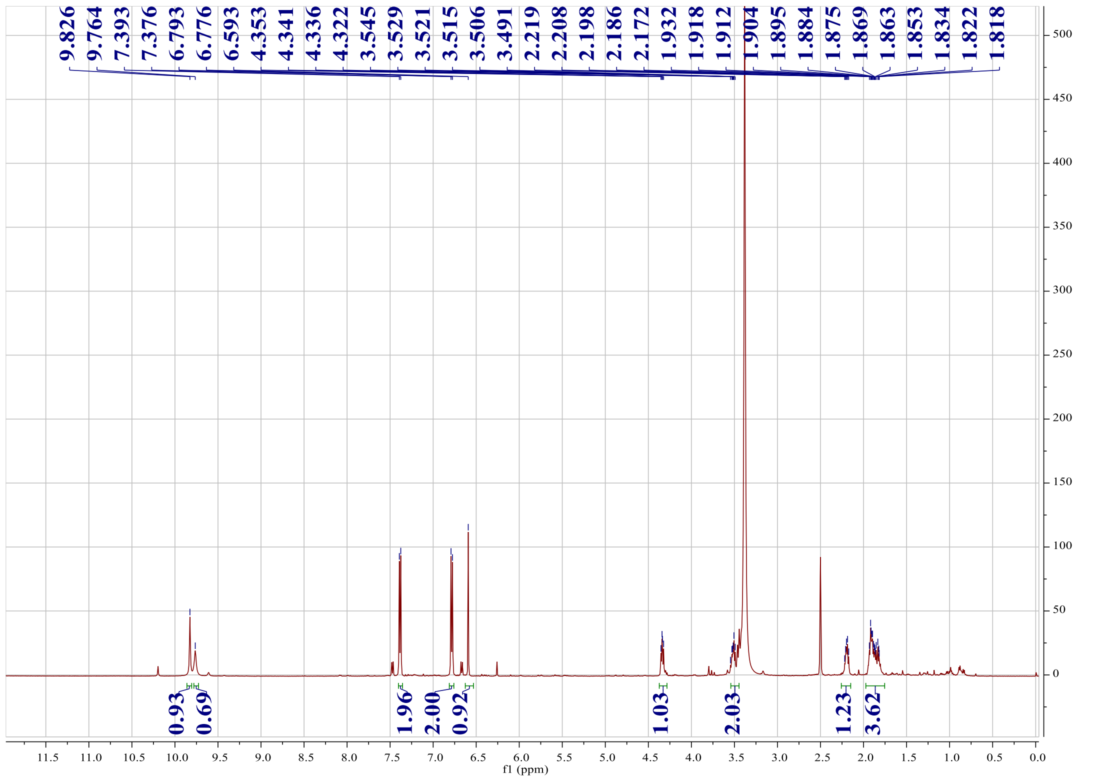

Figure S1. ^{1}H NMR (500 MHz) spectrum of **1** in DMSO-d_6.

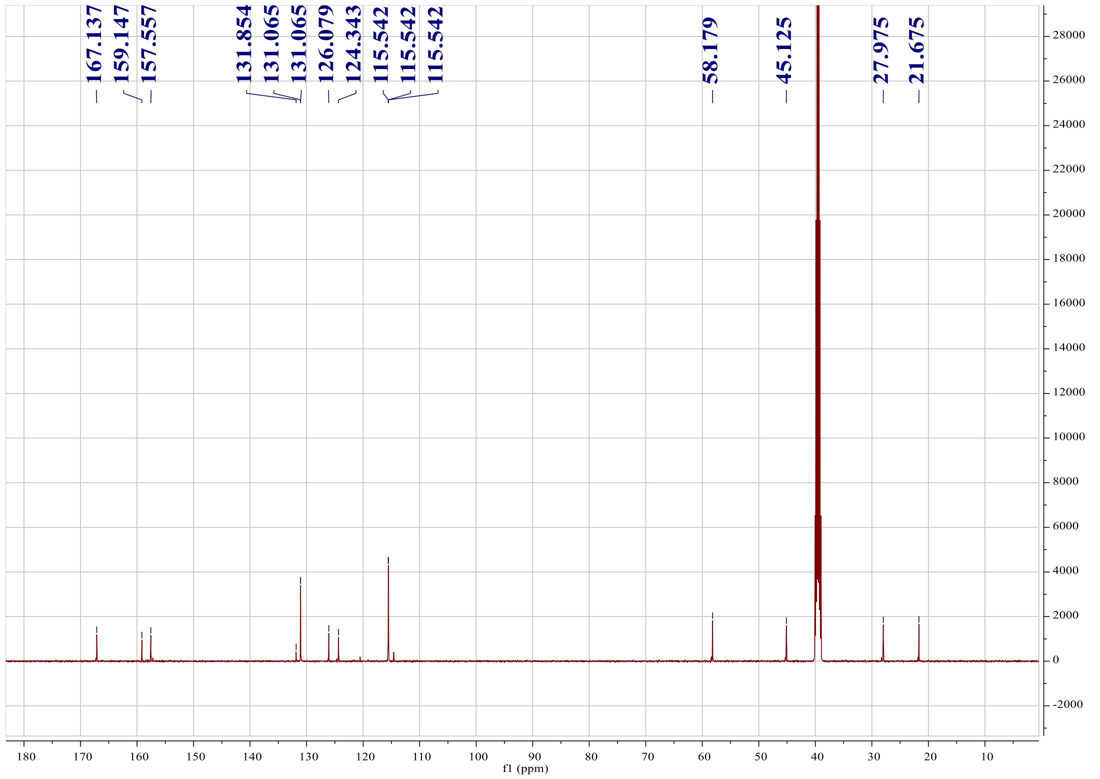

Figure S2. ^{13}C NMR (125 MHz) spectrum of **1** in DMSO-d_6.

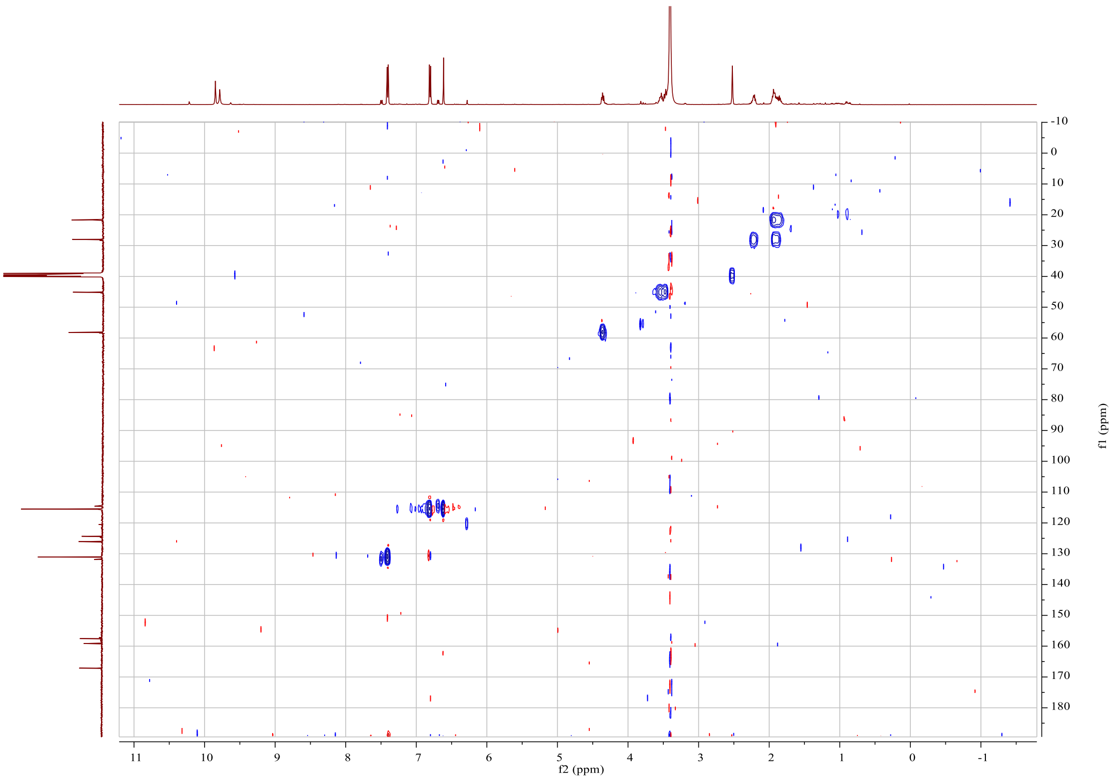

Figure S3. HSQC spectrum of **1** in DMSO-d_6.

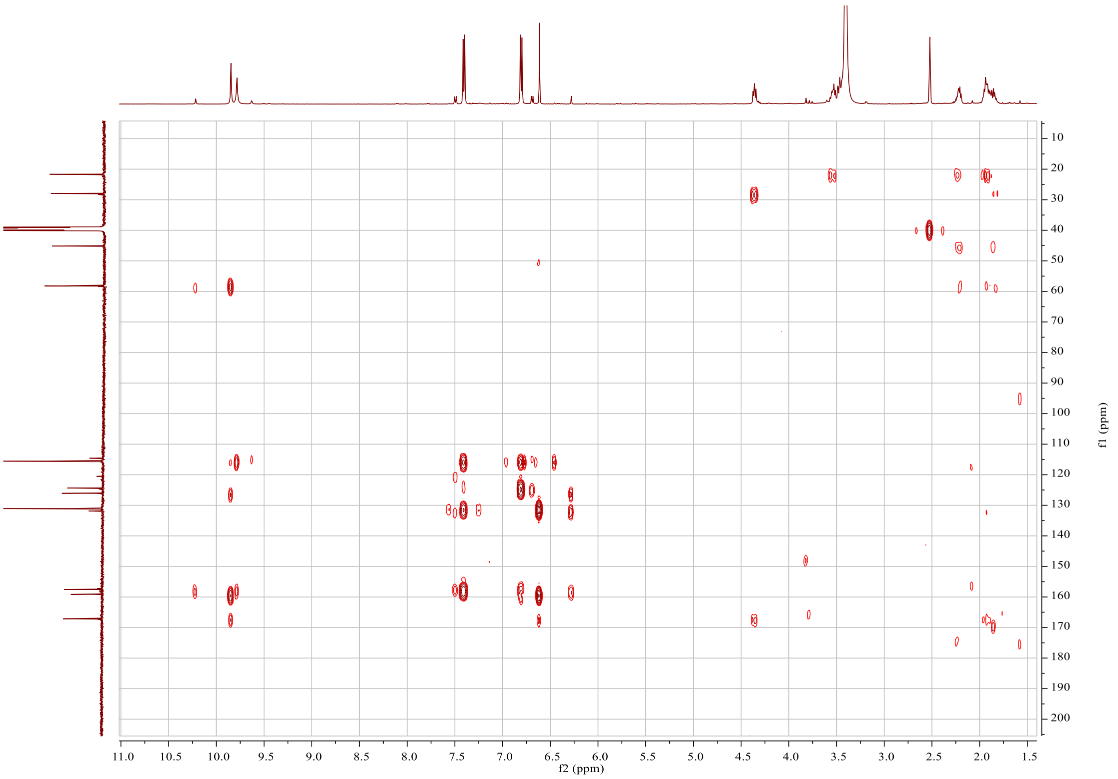

Figure S4. HMBC spectrum of **1** in DMSO-d_6.

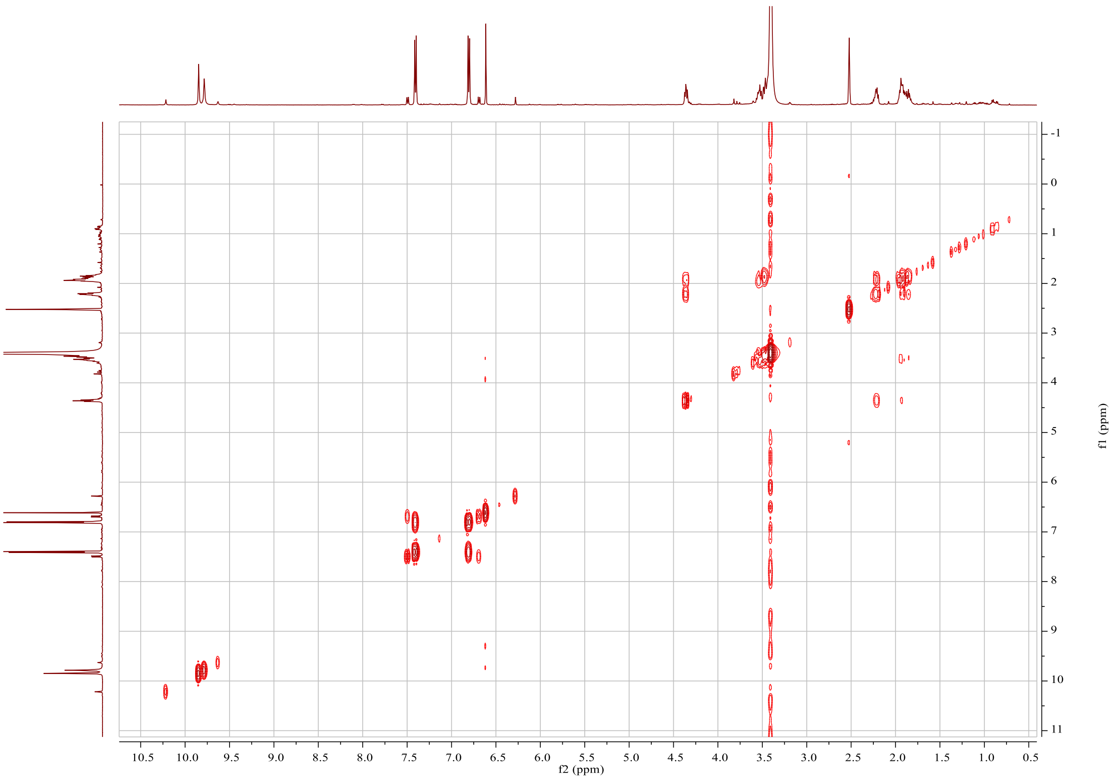

Figure S5. ^{1}H–^{1}H COSY spectrum of **1** in DMSO-d_6.

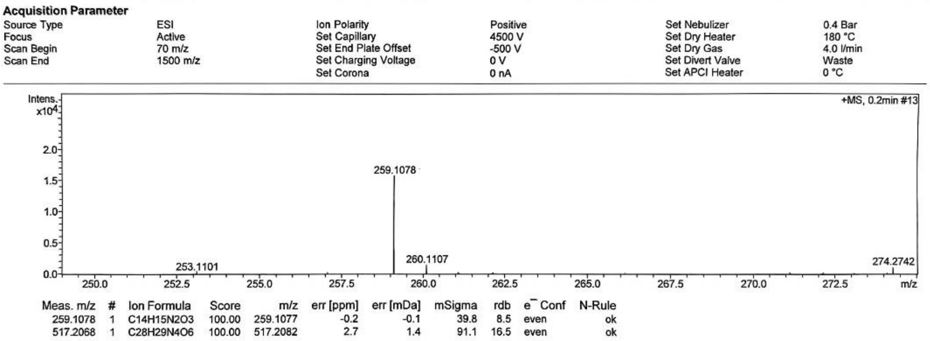

Figure S6. HRESIMS spectrum of **1**.

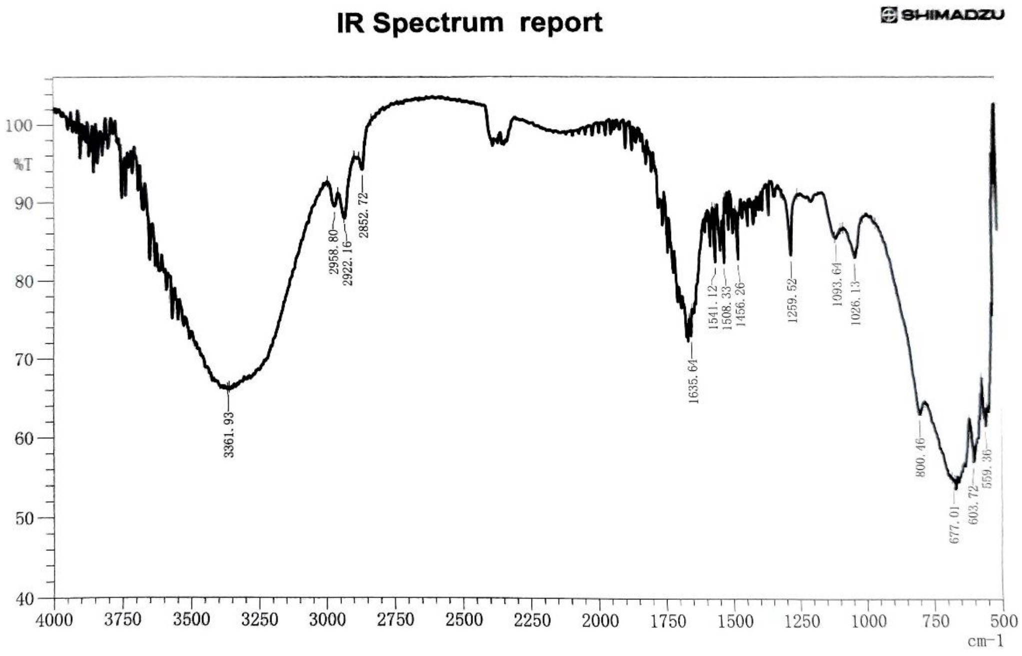

Figure S7. IR spectrum of **1**.

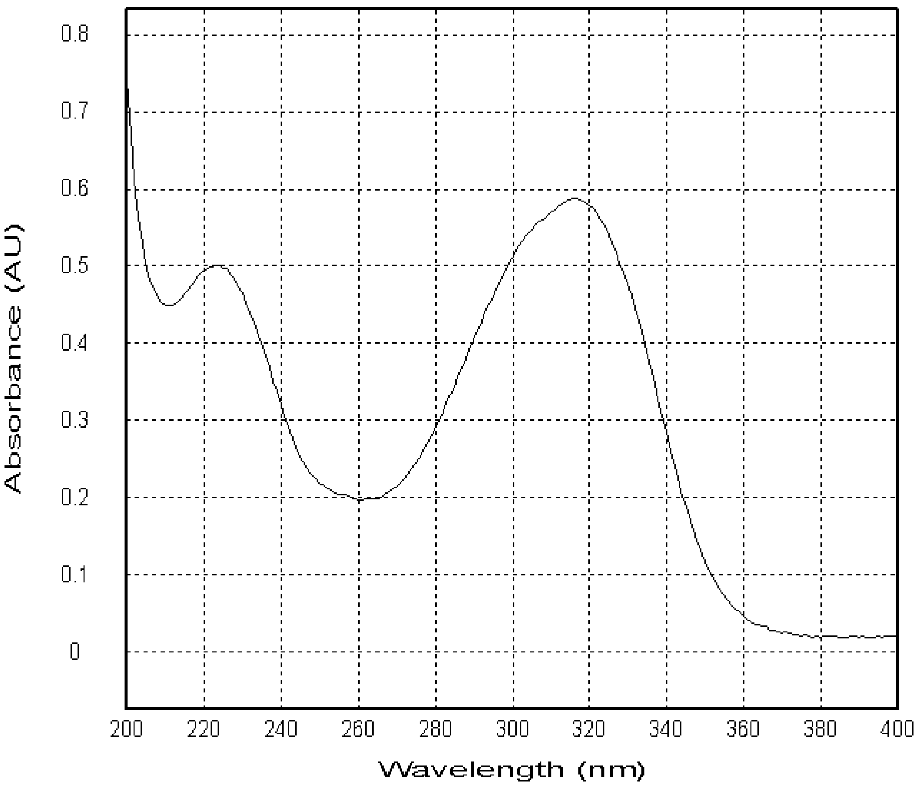

Figure S8. UV spectrum of **1** in MeOH.

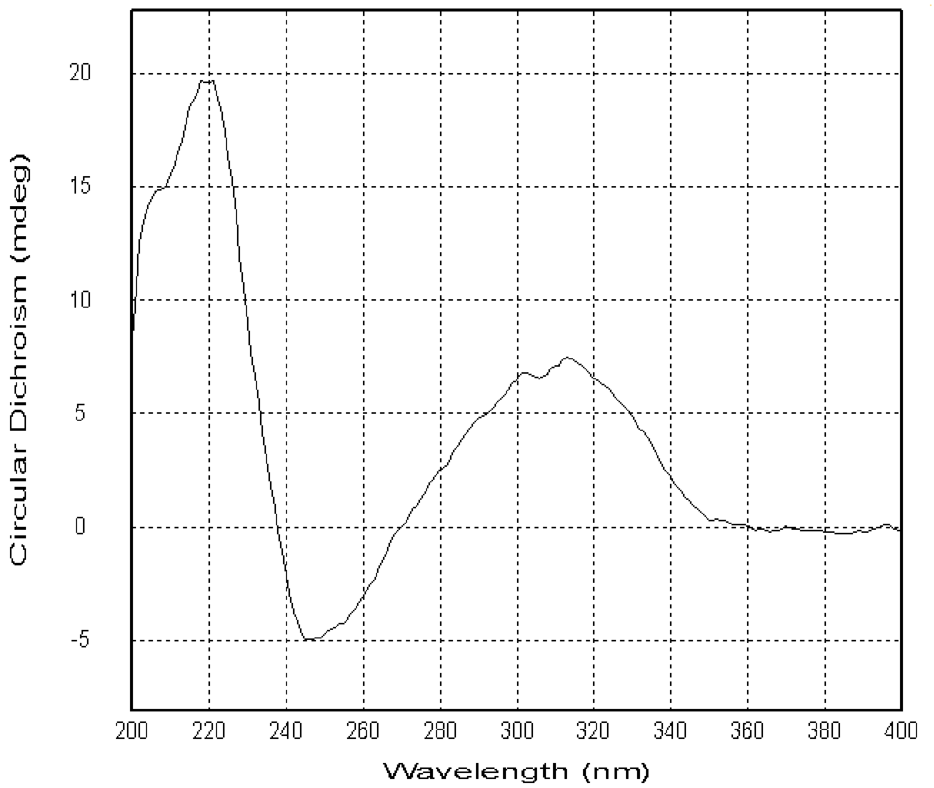

Figure S9. ECD spectrum of **1** in MeOH.

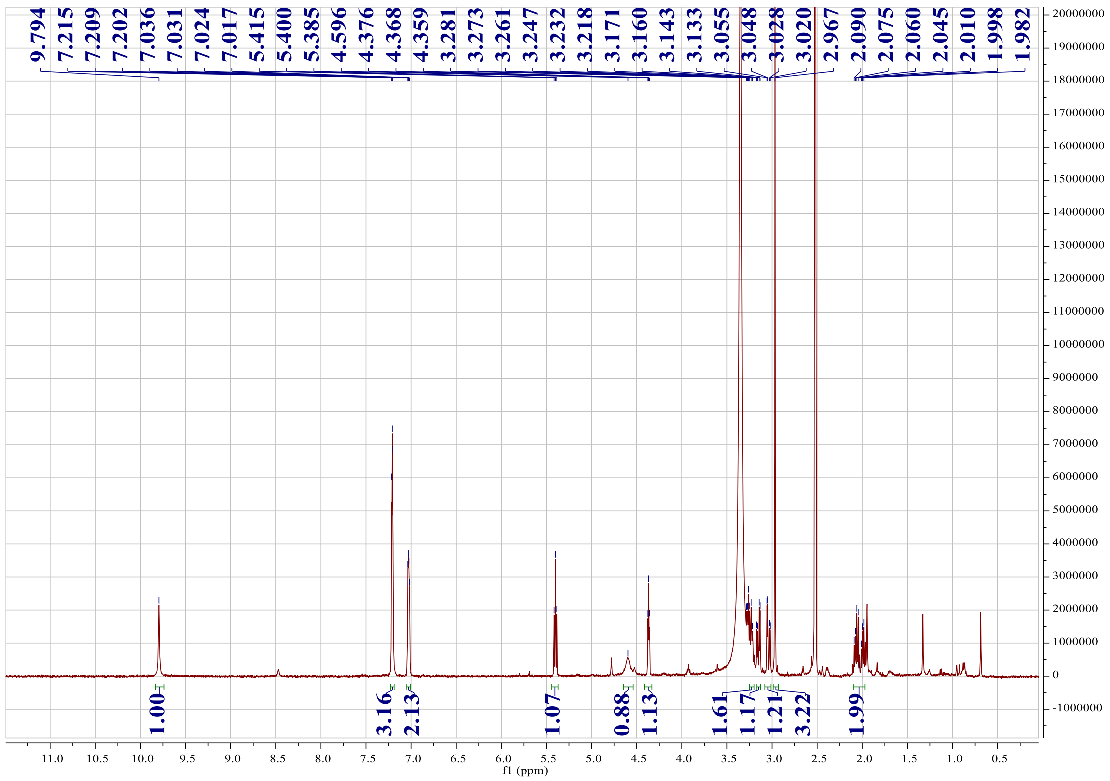

Figure S10. ^{1}H NMR (500 MHz) spectrum of **2** in DMSO-d_6.

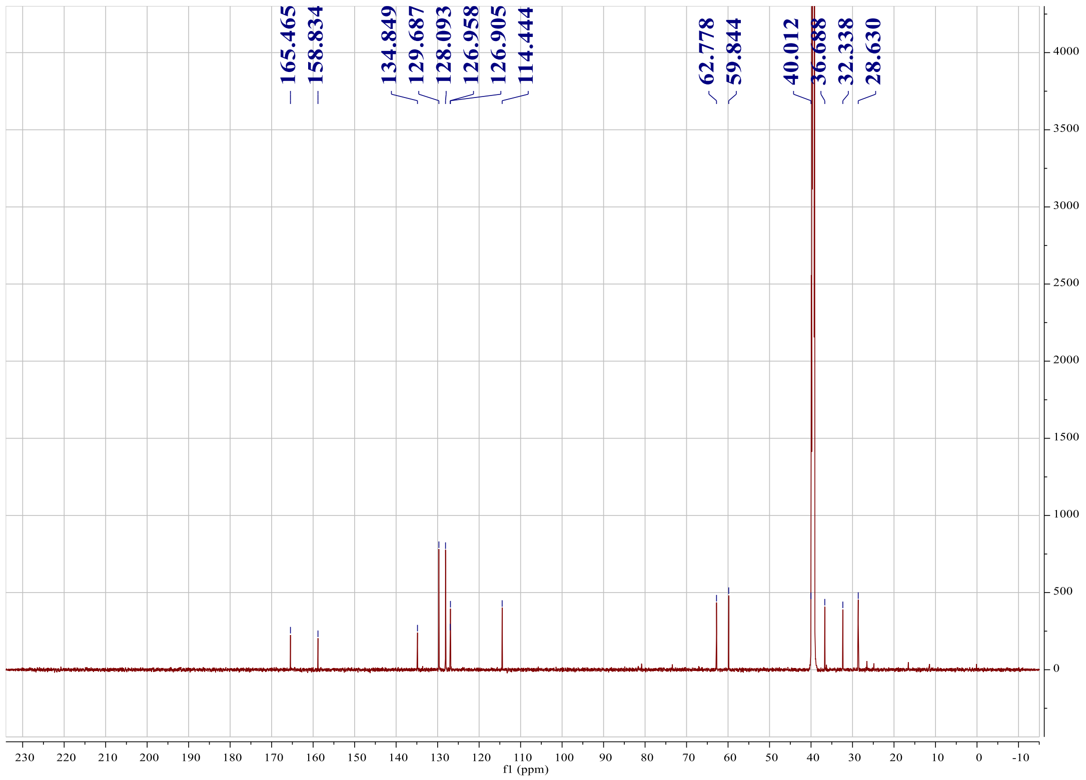

Figure S11. ^{13}C NMR (125 MHz) spectrum of **2** in DMSO-d_6.

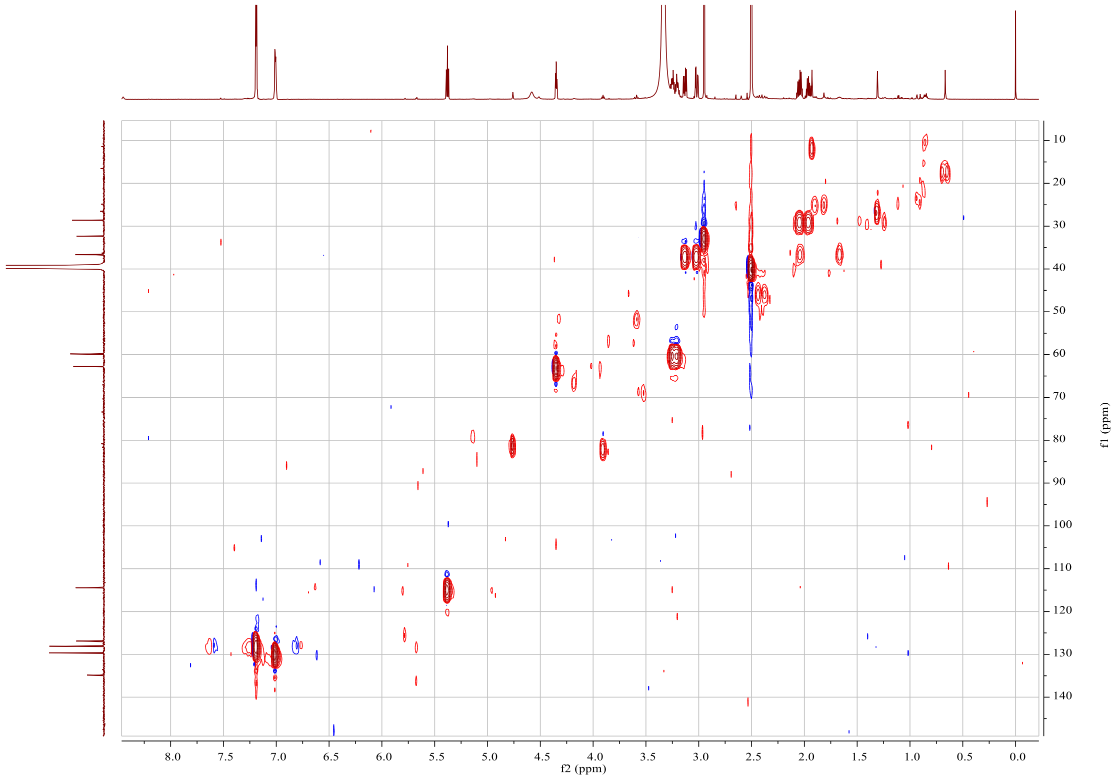

Figure S12. HSQC spectrum of **2** in DMSO-d_6.

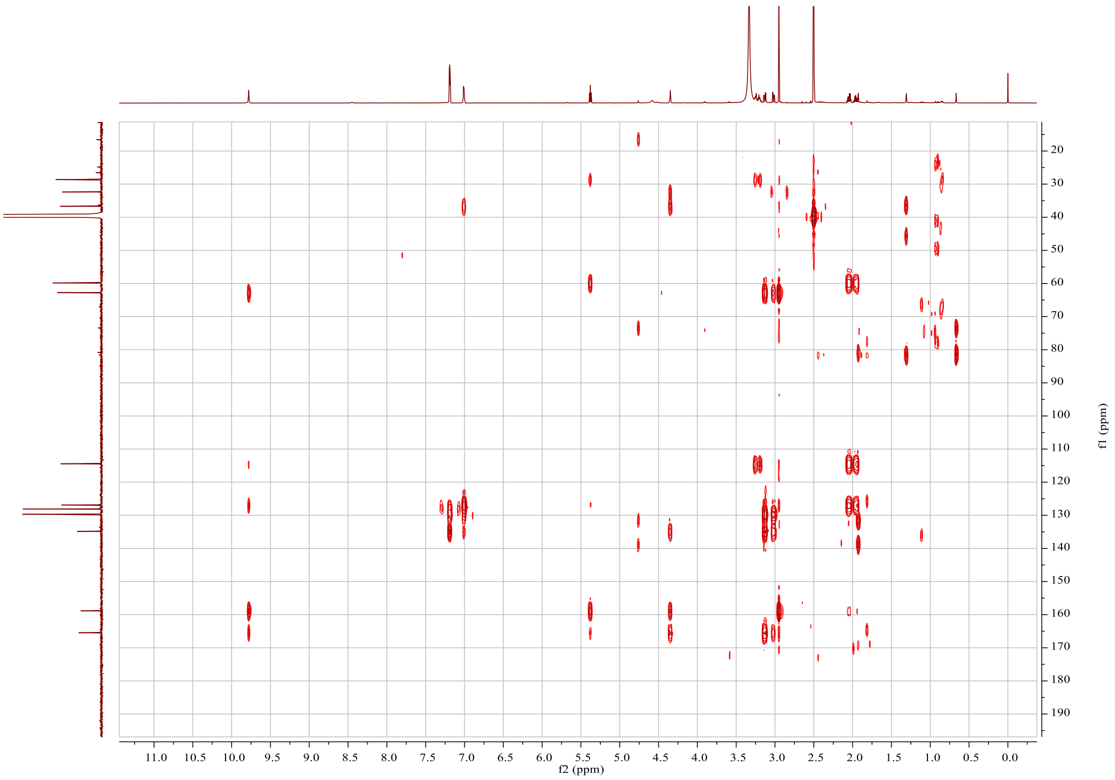

Figure S13. HMBC spectrum of **2** in DMSO-*d*6.

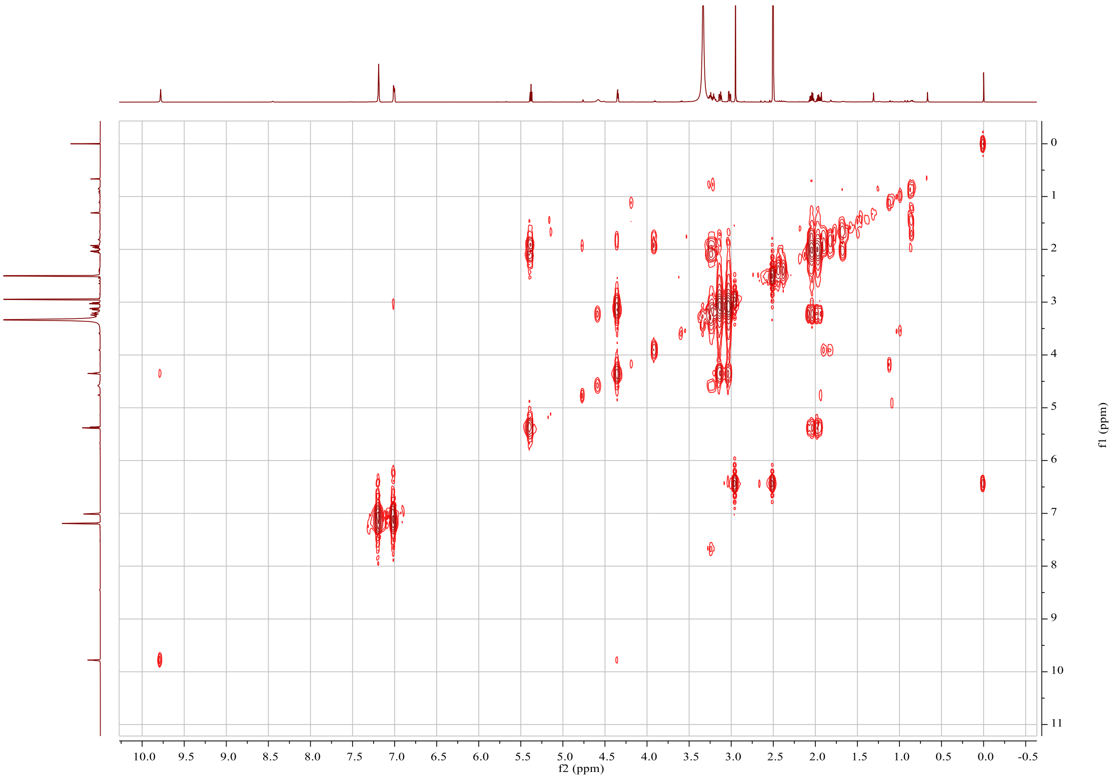

Figure S14. ^{1}H–^{1}H COSY spectrum of **2** in DMSO-*d*6.

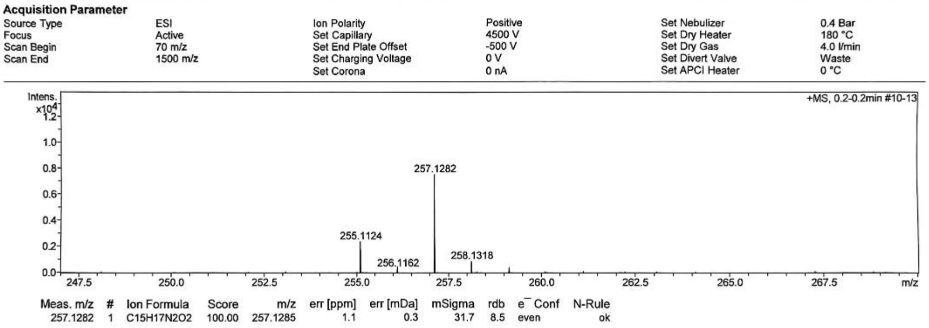

Figure S15. HRESIMS spectrum of **2**.

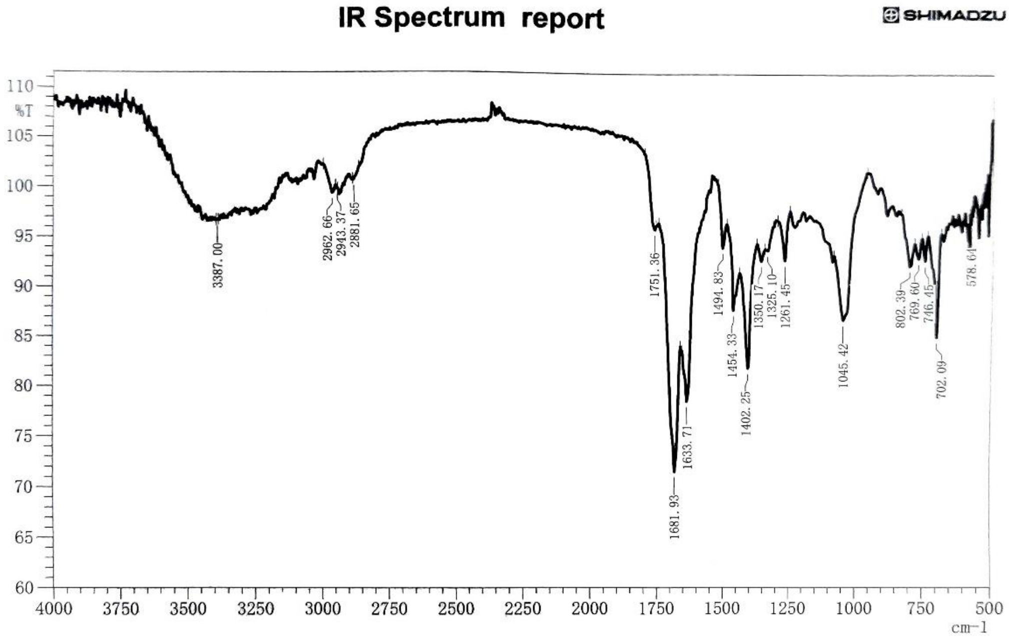

Figure S16. IR spectrum of **2**.

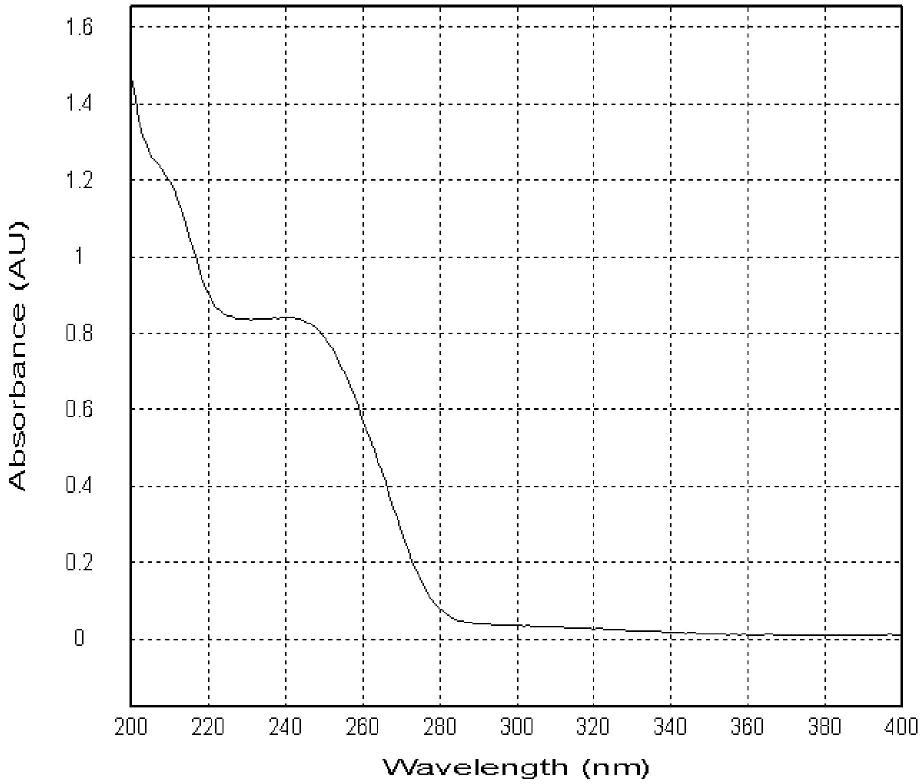

Figure S17. UV spectrum of **2** in MeOH.

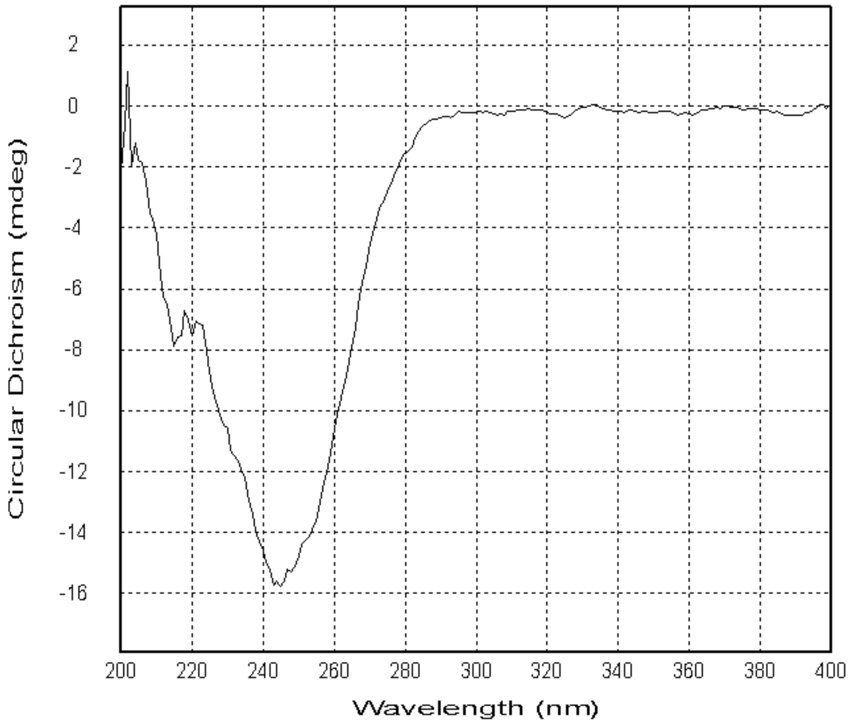

Figure S18. ECD spectrum of **2** in MeOH.

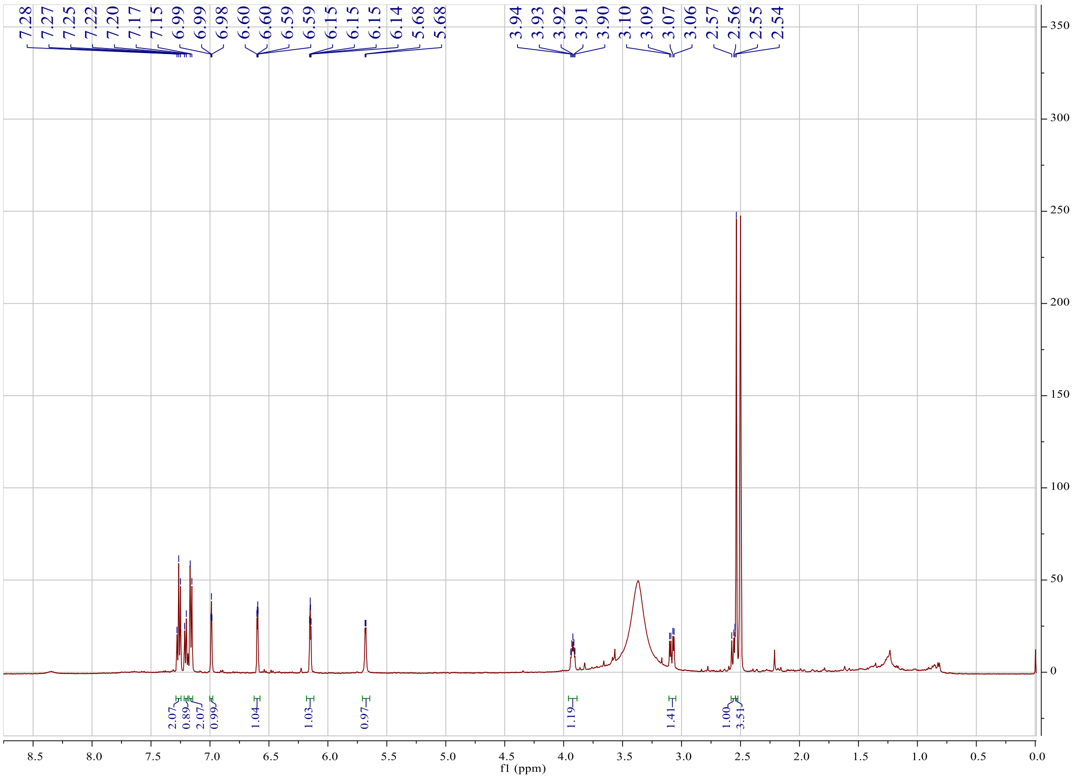

Figure S19. ^{1}H NMR (500 MHz) spectrum of **3** in DMSO-d_6.

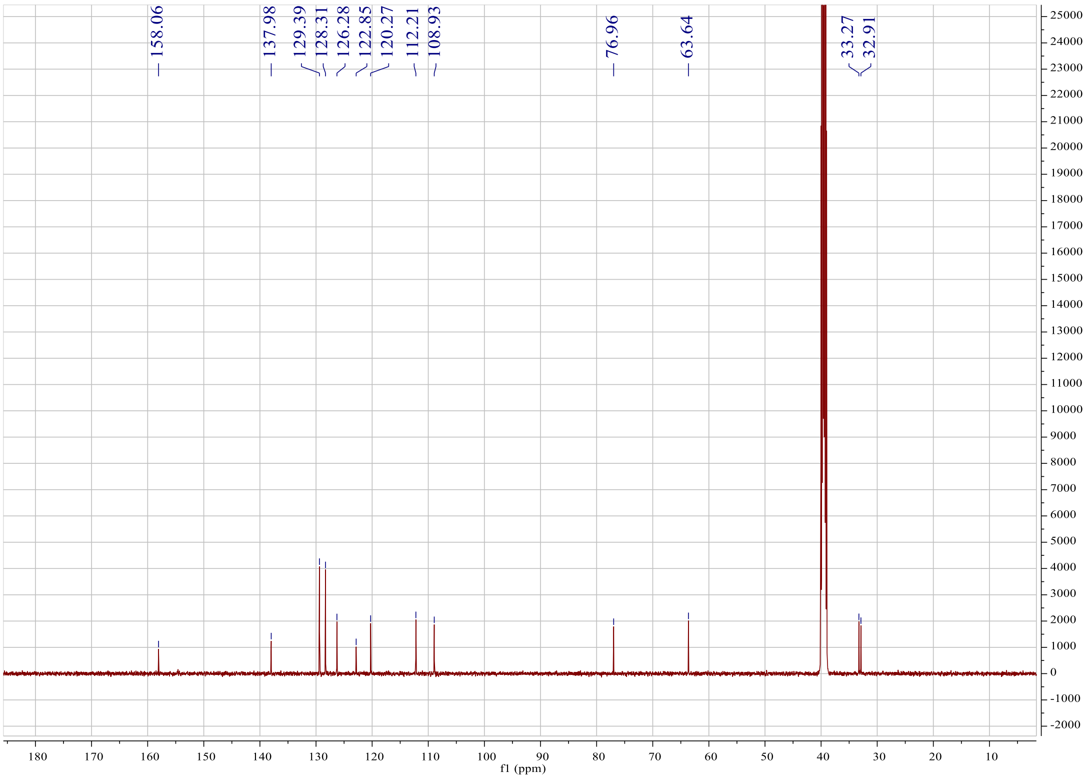

Figure S20. ^{13}C NMR (125 MHz) spectrum of **3** in DMSO-d_6.

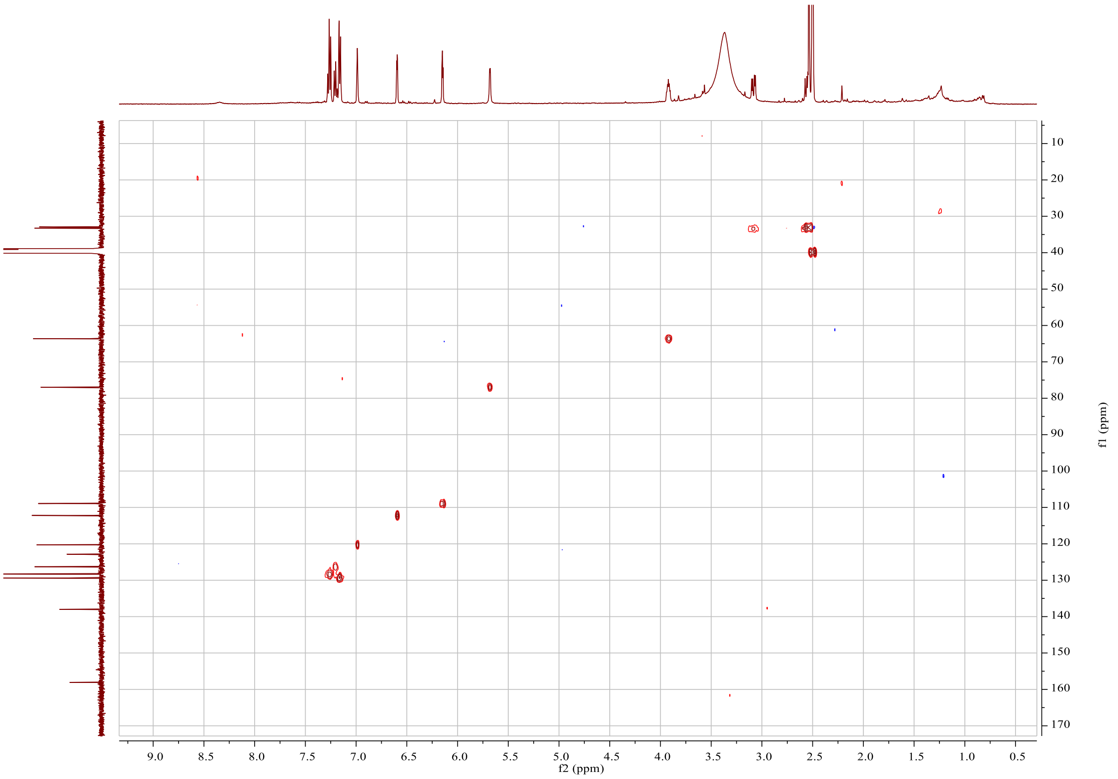

Figure S21. HSQC spectrum of **3** in DMSO-d_6.

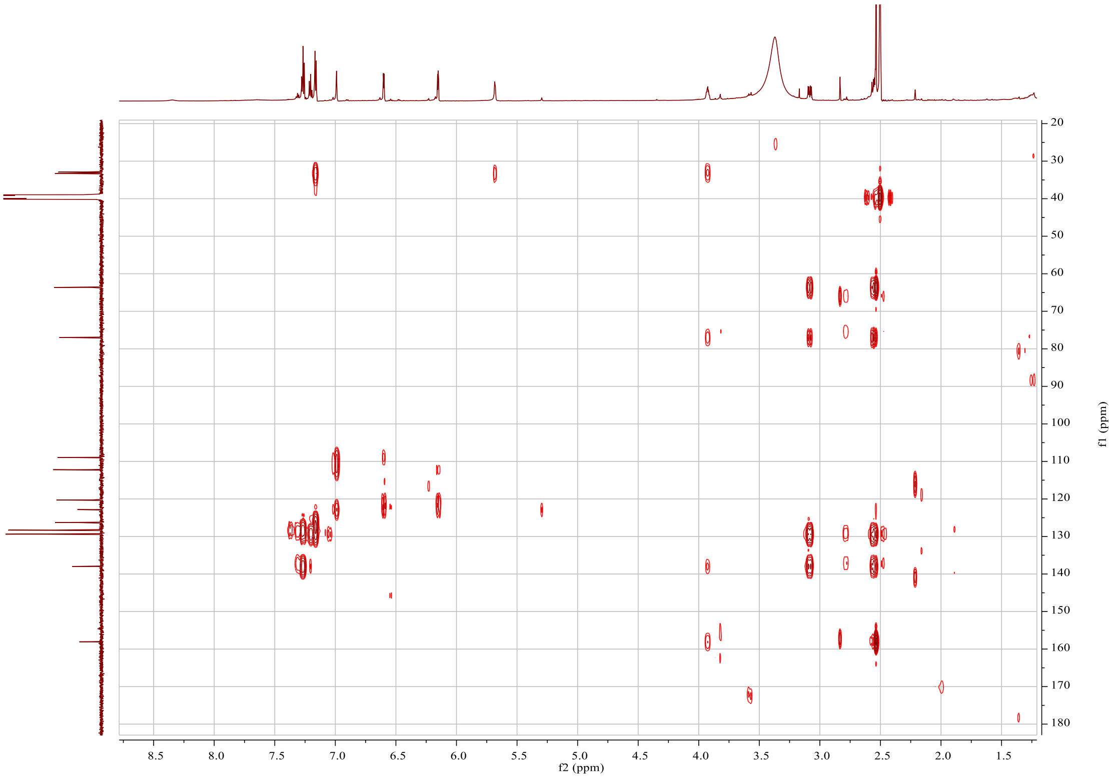

Figure S22. HMBC spectrum of **3** in DMSO-d_6.

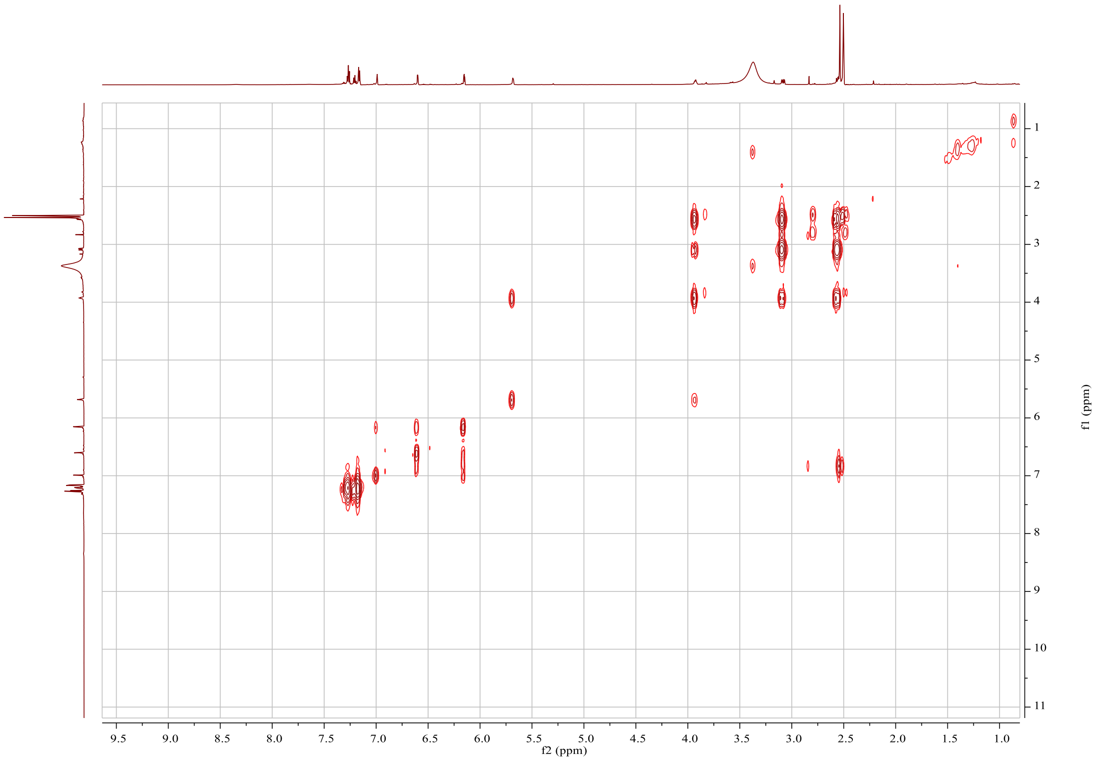

Figure S23. ^{1}H–^{1}H COSY spectrum of **3** in DMSO-d_6.

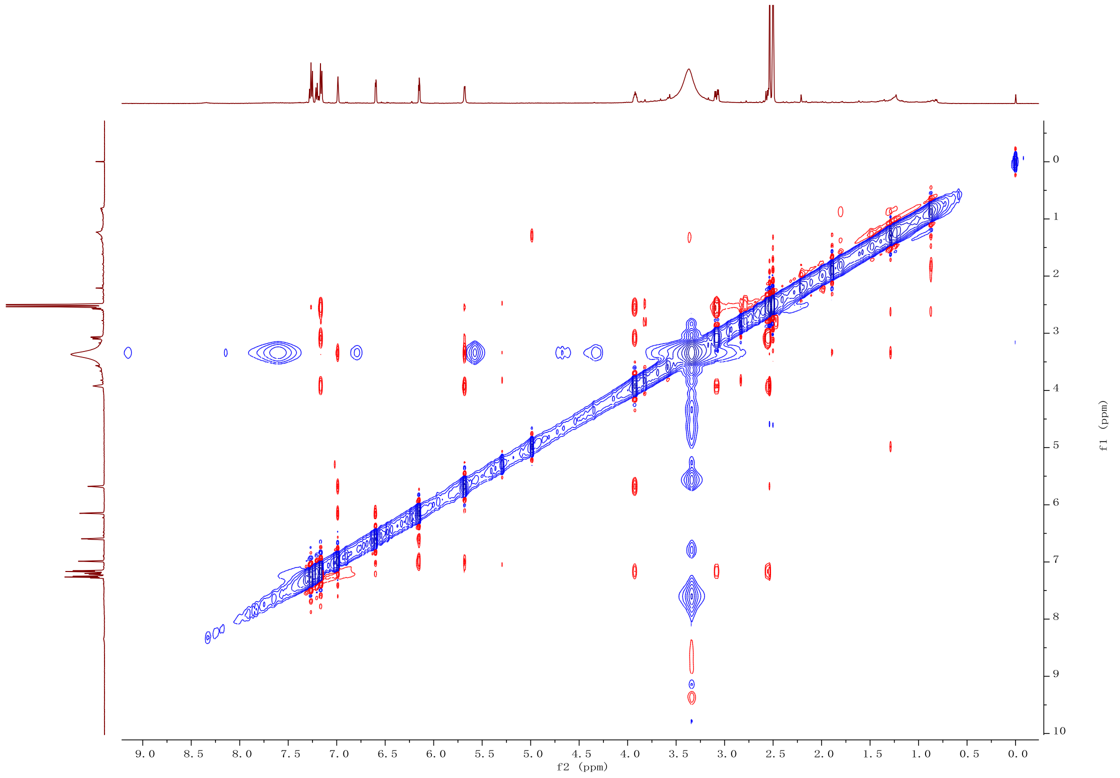

Figure S24. NOESY spectrum of **3** in DMSO-d_6.

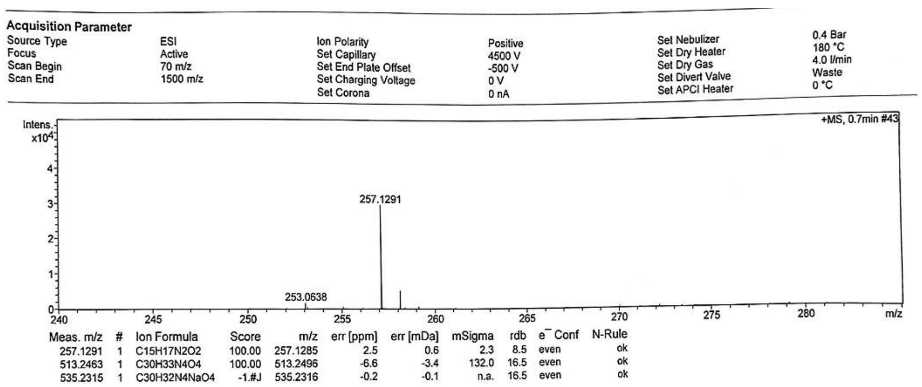

Figure S25. HRESIMS spectrum of **3**.

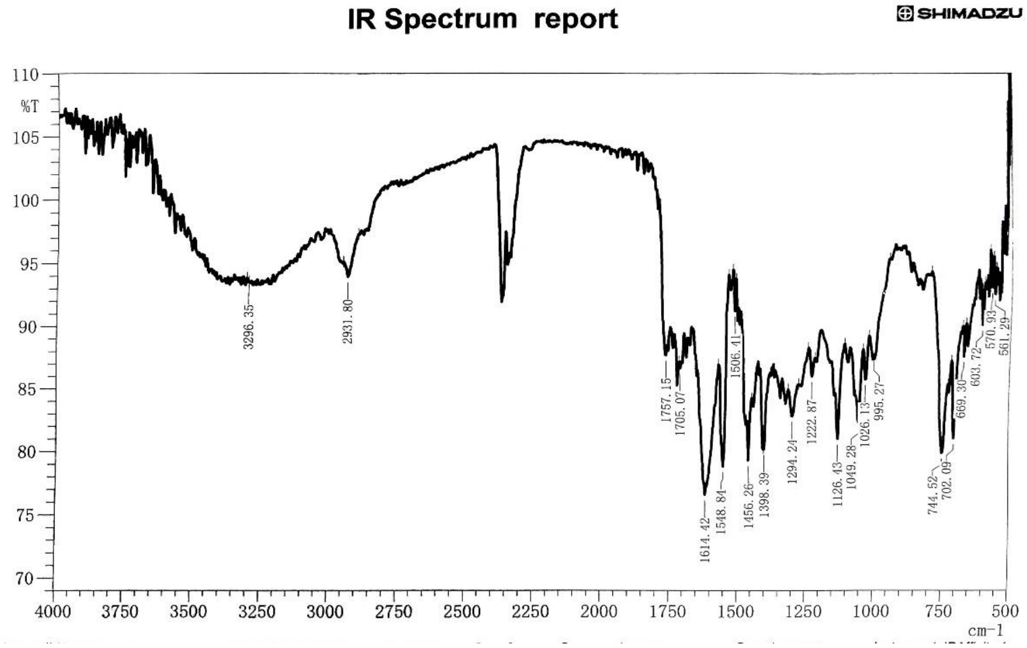

Figure S26. IR spectrum of **3**.

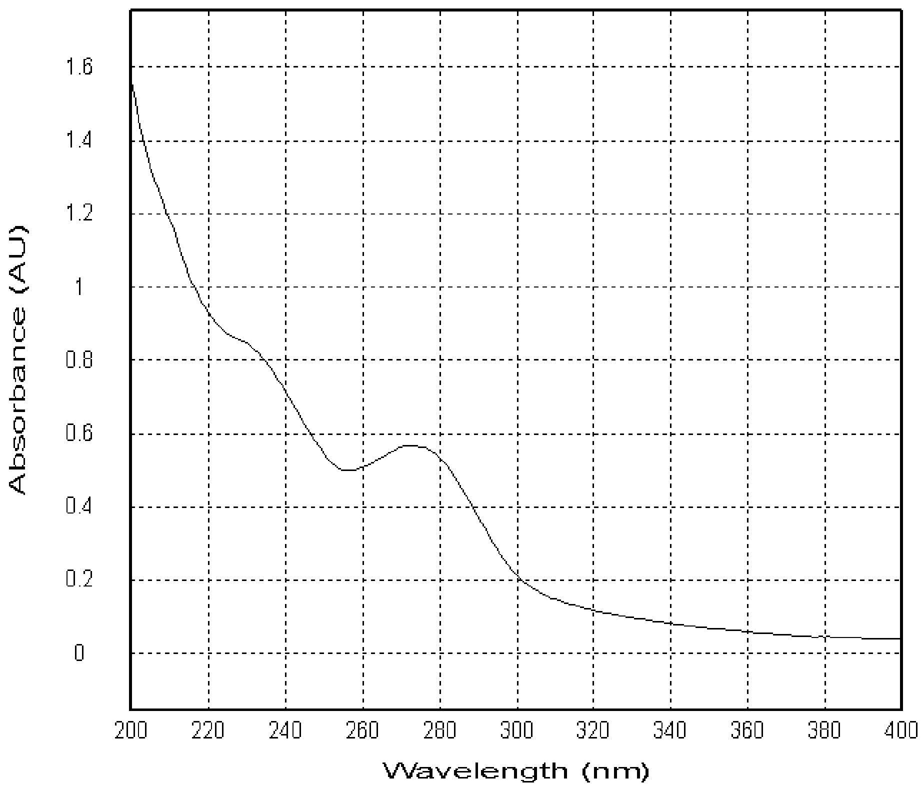

Figure S27. UV spectrum of **3** in MeOH.

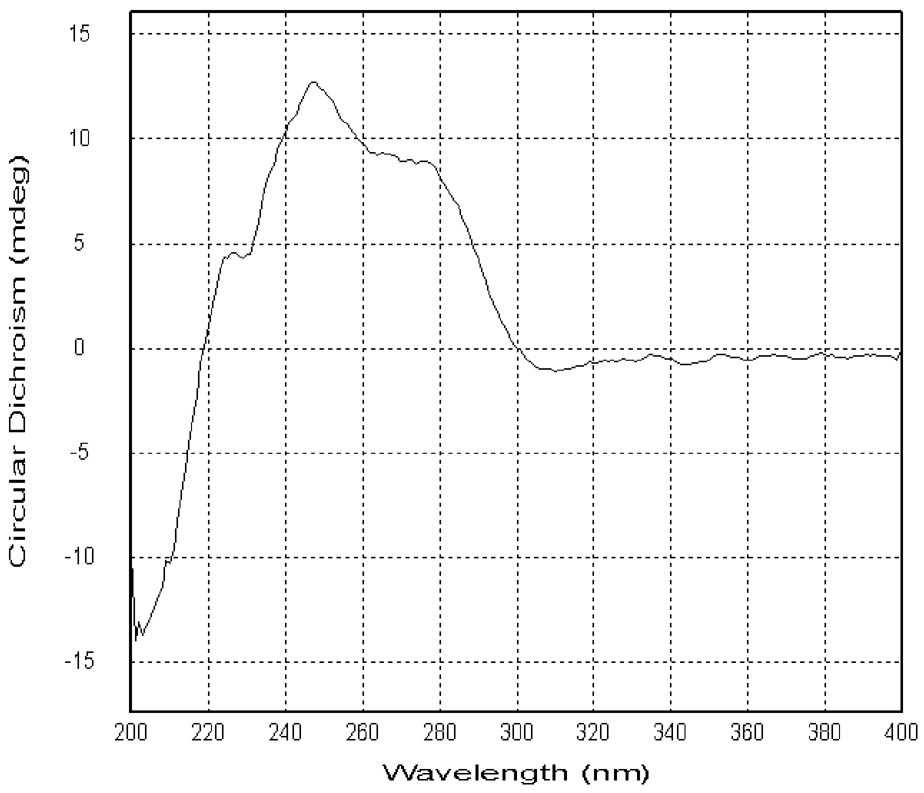

Figure S28. ECD spectrum of **3** in MeOH.

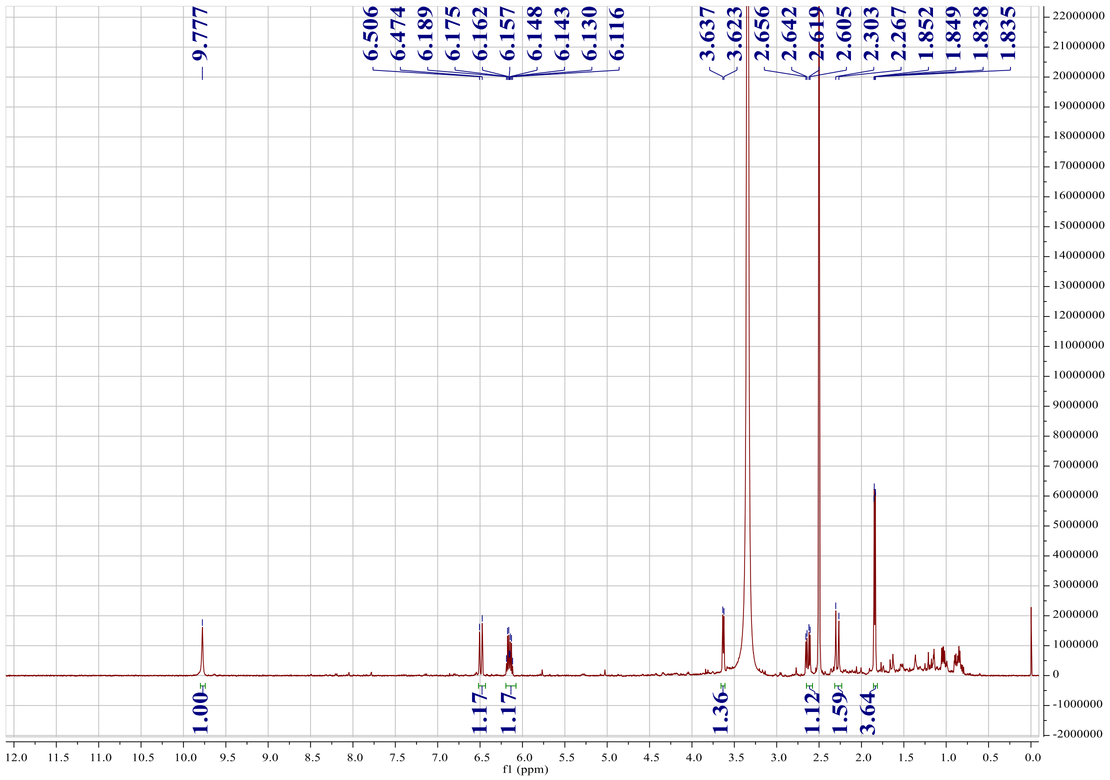

Figure S29. ^{1}H NMR (500 MHz) spectrum of **10** in DMSO-d_6.

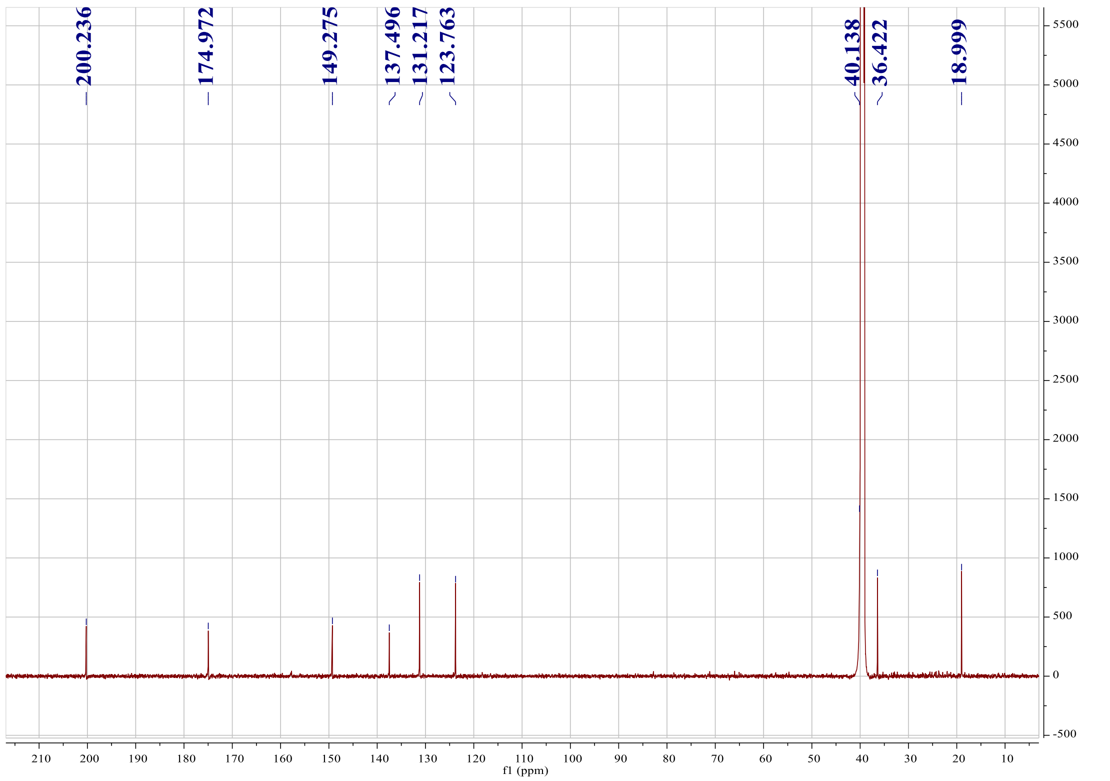

Figure S30. ^{13}C NMR (125 MHz) spectrum of **10** in DMSO-d_6.

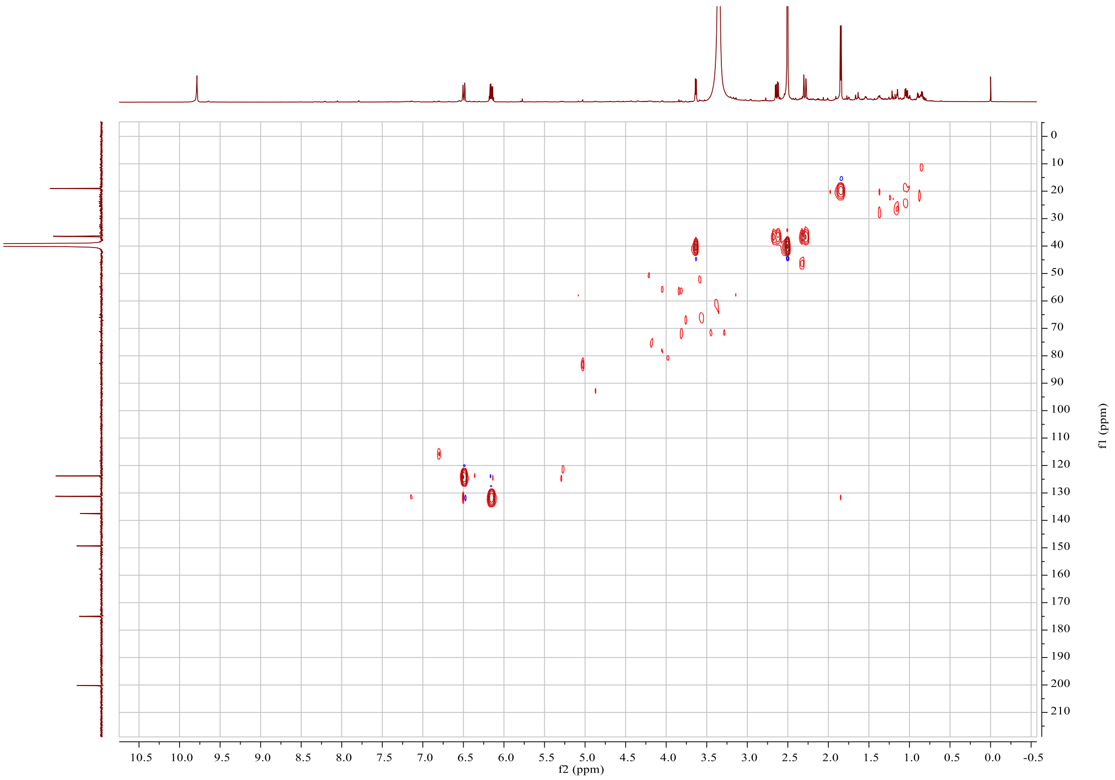

Figure S31. HSQC spectrum of **10** in DMSO-d_6.

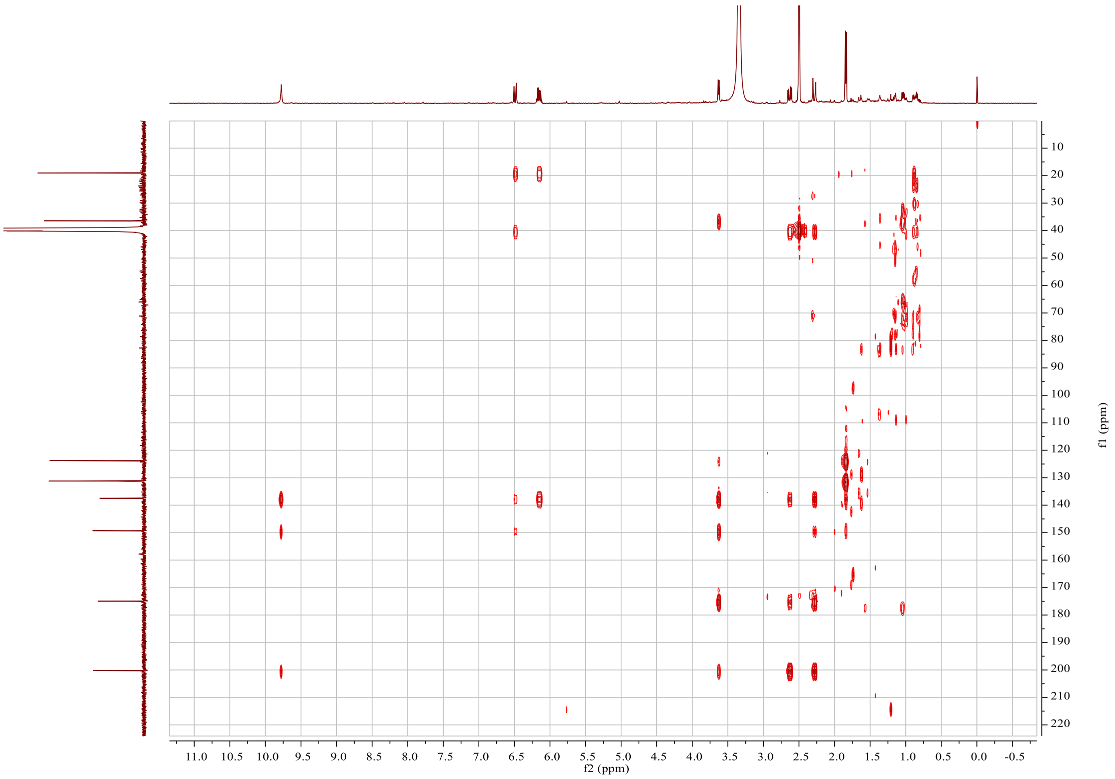

Figure S32. HMBC spectrum of **10** in DMSO-d_6.

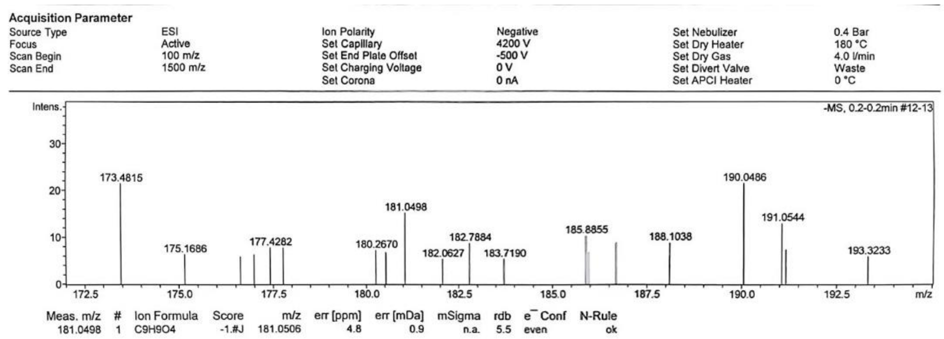

Figure S33. HRESIMS spectrum of **10**.

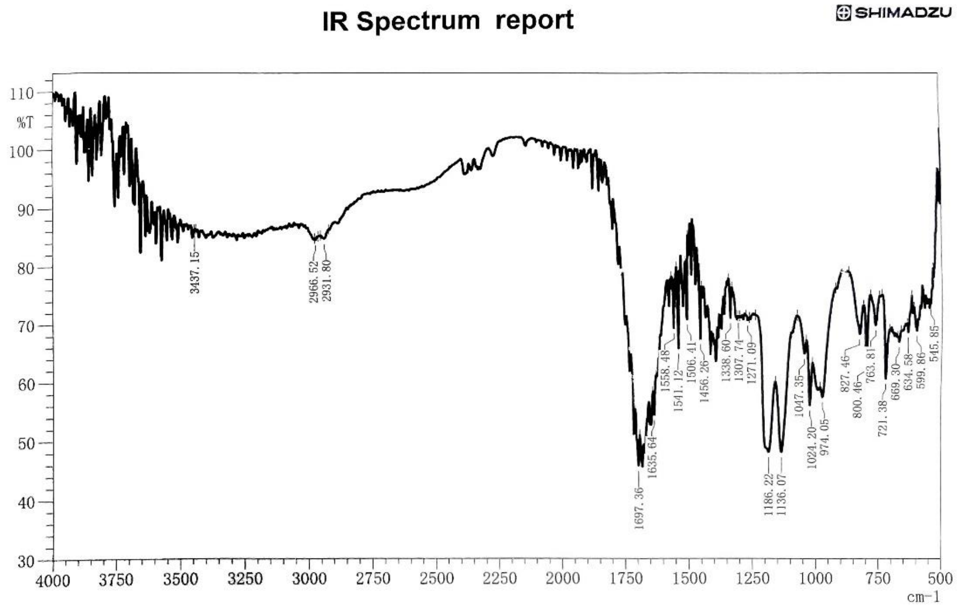

Figure S34. IR spectrum of **10**.

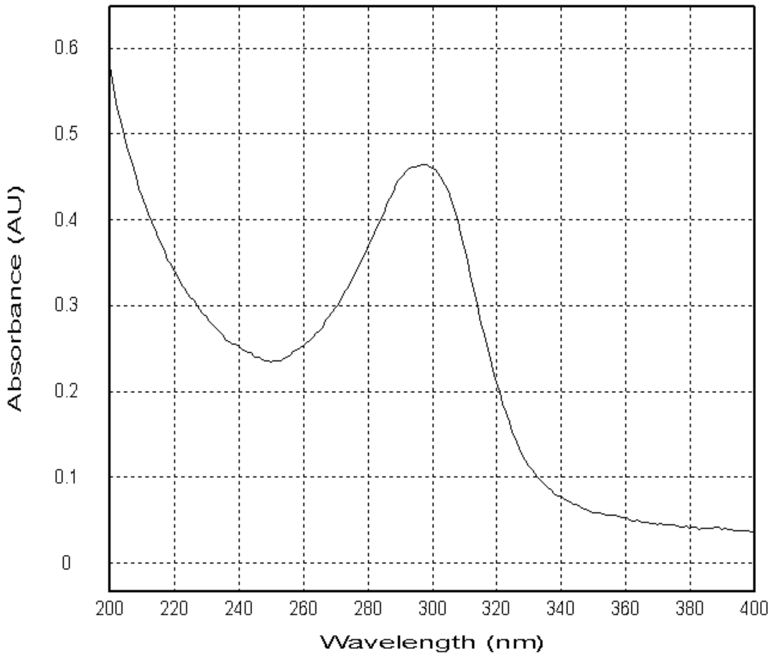

Figure S35. UV spectrum of **10** in MeOH.

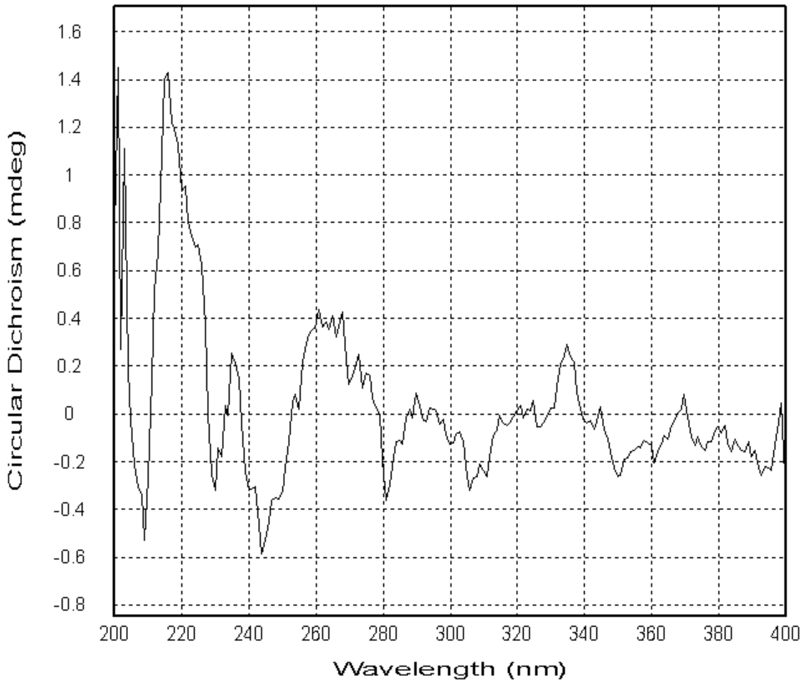

Figure S36. ECD spectrum of **10** in MeOH.

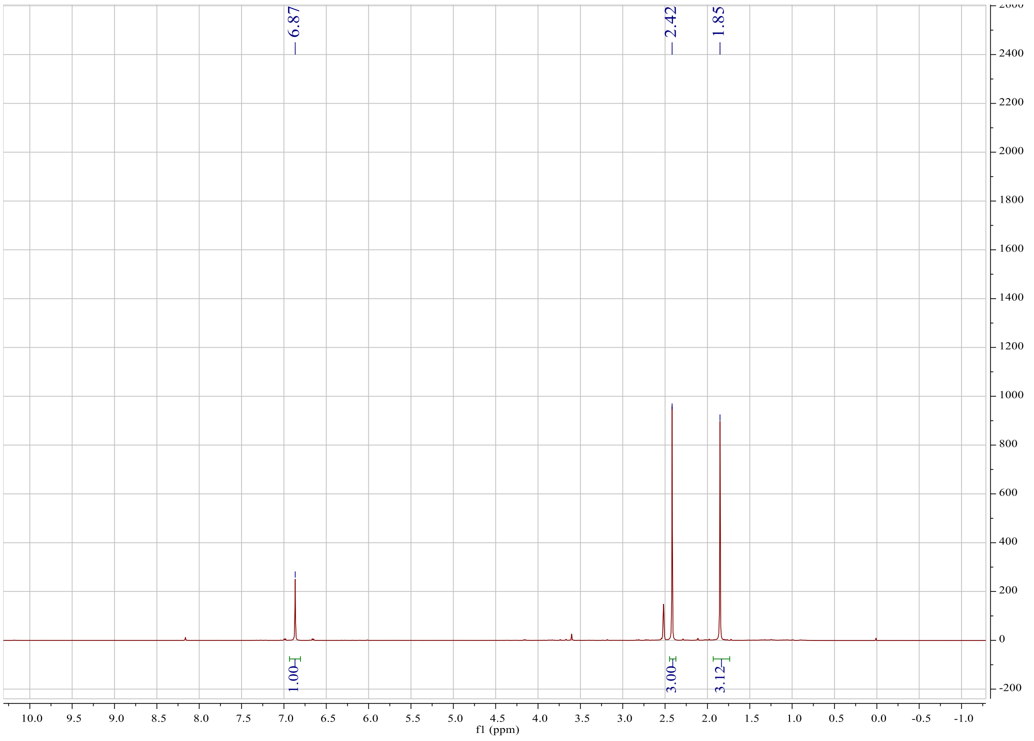

Figure S37. ^{1}H NMR (500 MHz) spectrum of **11** in DMSO-d_6.

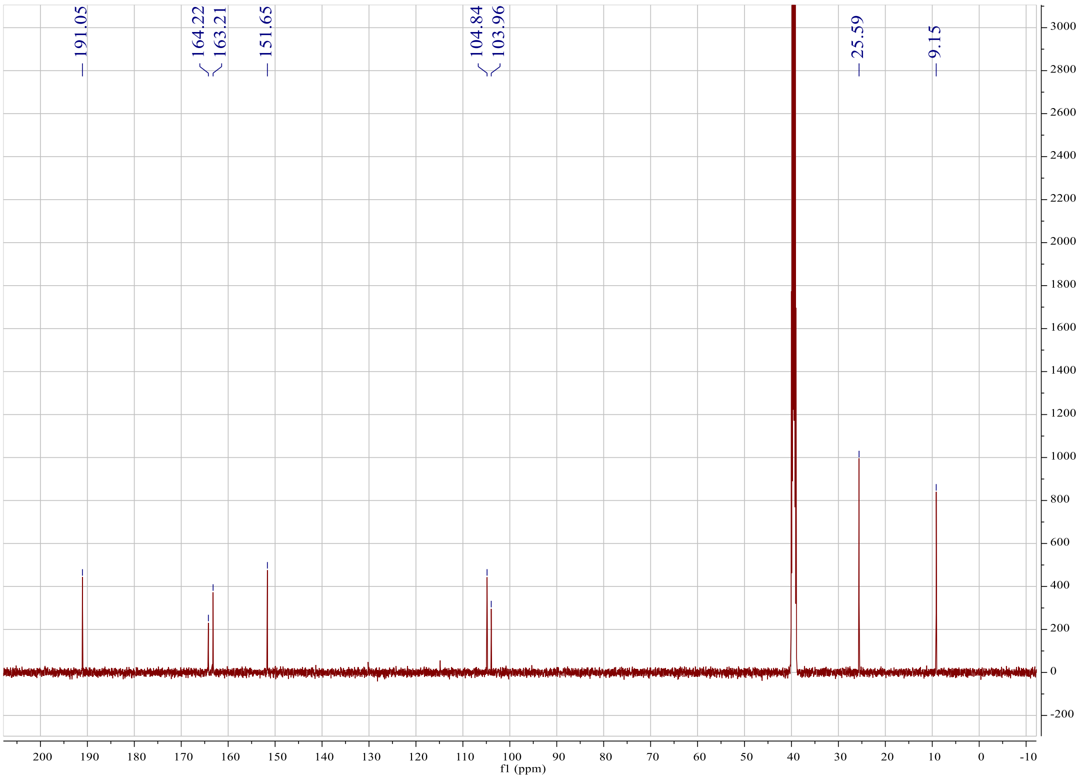

Figure S38. ^{13}C NMR (125 MHz) spectrum of **11** in DMSO-d_6.

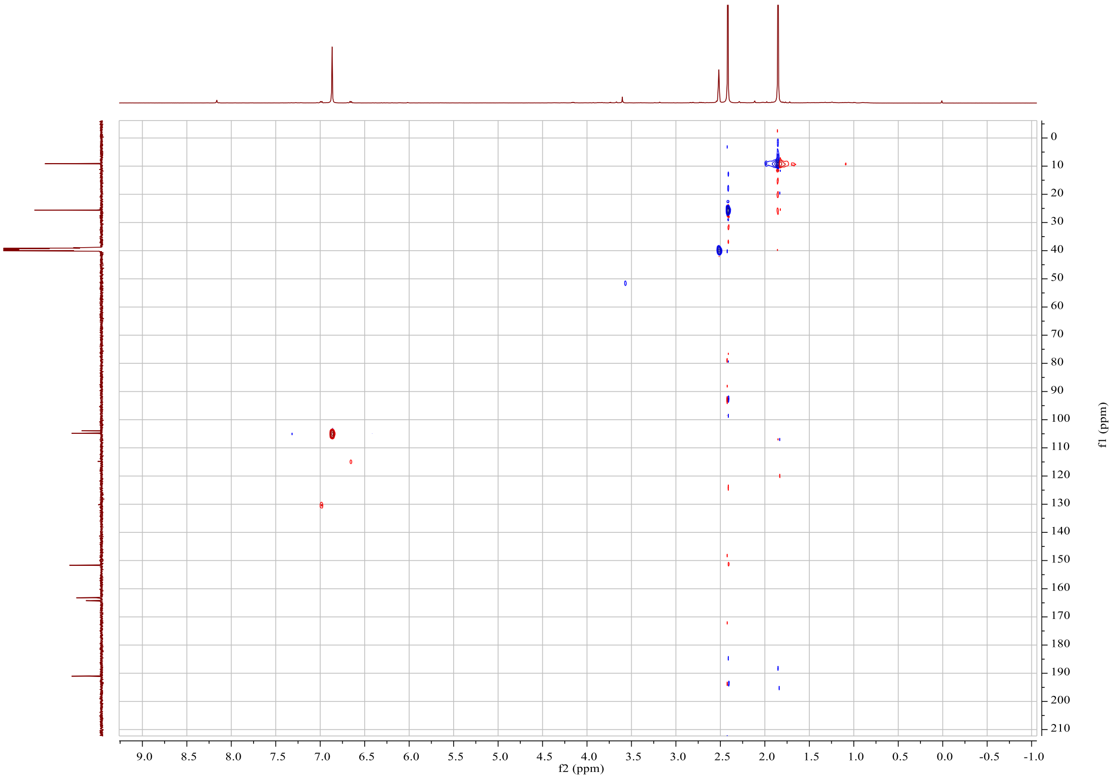

Figure S39. HMBC spectrum of **11** in DMSO-*d*6.

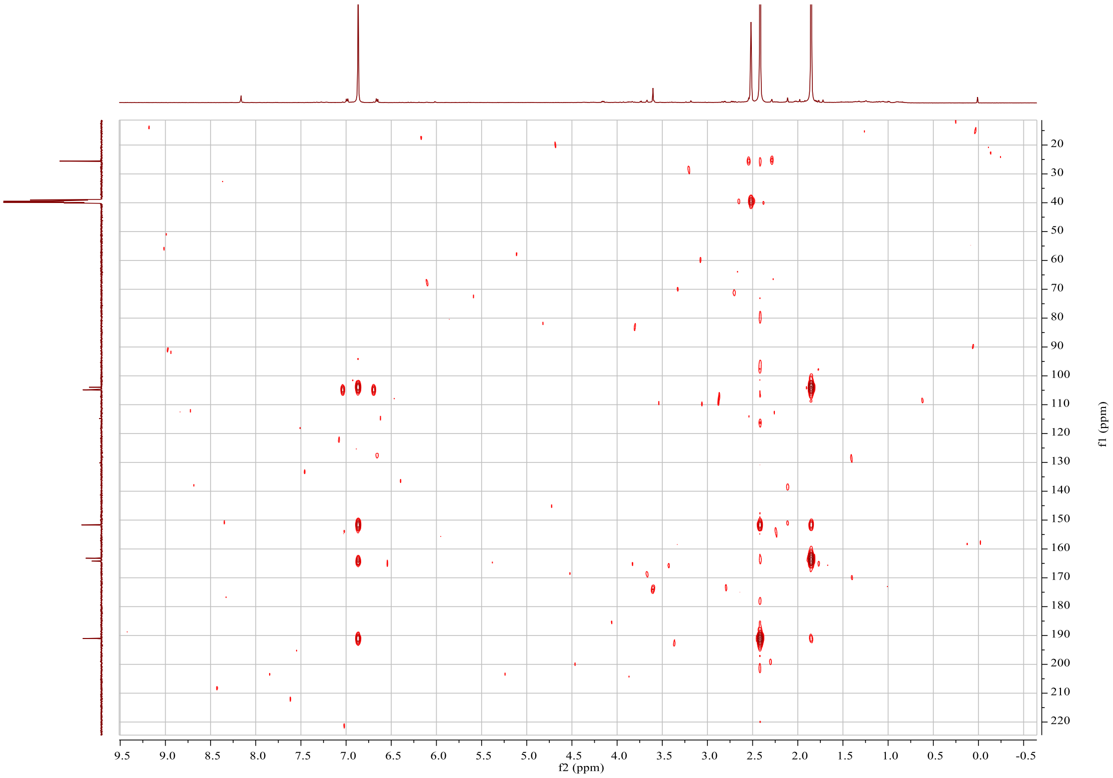

Figure S40. HSQC spectrum of **11** in DMSO-*d*6.

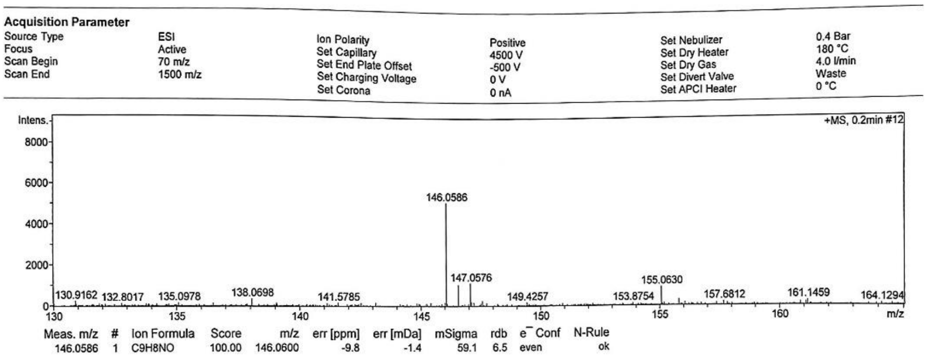

Figure S41. HRESIMS spectrum of **11**.

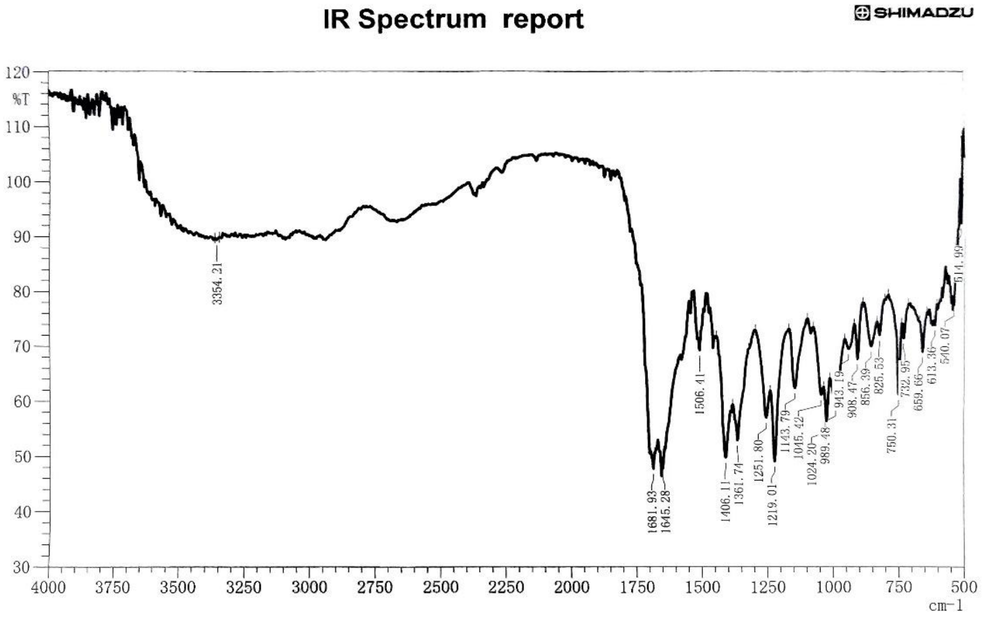

Figure S42. IR spectrum of **11**.

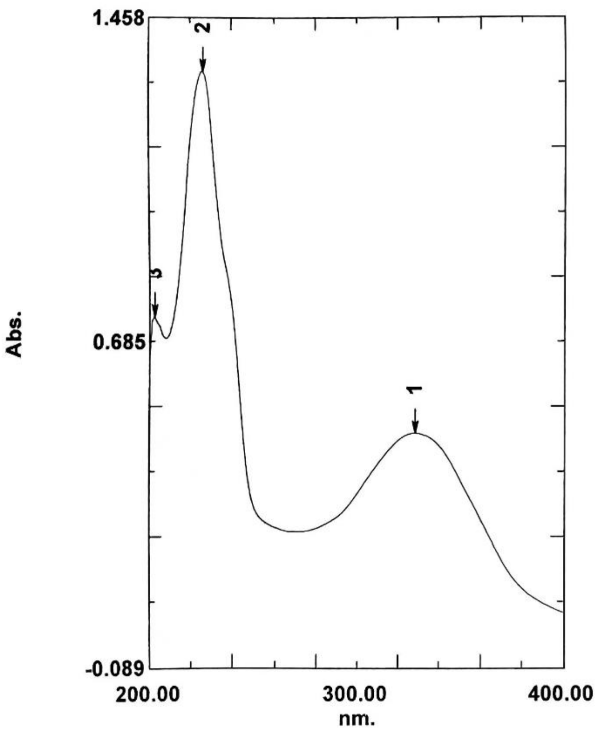

No.	P/V	波长(nm)	Abs.	描述
1		328.00	0.465	
2		226.00	1.329	
3		202.40	0.743	

Figure S43. UV spectrum of **11** in MeOH.

The physicochemical data of the known compounds

Cyclo(D-6-Hyp-L-Phe) (**4**): ^{1}H NMR (500 MHz, DMSO-d_6) δ 8.01 (1H, s), 7.25 (4H, m), 7.18 (1H, m), 6.56 (1H, s), 6.68 (1H, s), 4.42 (1H, t, J = 4.9 Hz), 3.45 (1H, m), 3.07 (1H, m), 1.92 (2H, m) , 1.72 (1H, m) , 1.65 (1H, t, J = 9.1 Hz); ^{13}C NMR (125 MHz, DMSO-d_6) δ 167.8 (C), 166.0 (C), 137.0 (C), 129.9 (CH), 127.9 (CH), 126.4 (CH), 86.6 (C), 55.5 (CH), 44.3 (CH_2), 35.6 (CH_2), 35.4 (CH_2), 19.4 (CH_2).

Cyclo(l-6-Hyp-L-Phe) (**5**): ^{1}H NMR (500 MHz, DMSO-d_6) δ 8.04 (1H, d, J = 4.6 Hz), 7.31 (1H, t, J = 7.4 Hz), 7.25 (1H, m), 7.20 (1H, m), 6.68 (1H, s), 3.84 (1H, m), 3.50 (1H, m), 3.42 (1H, m), 3.13 (1H, m) , 2.04 (1H, m) , 1.84 (1H, d, J = 6.3 Hz); ^{13}C NMR (125 MHz, DMSO-d_6) δ 167.8 (C), 166.9 (C), 137.4 (C), 129.4 (CH), 128.3 (CH), 126.5 (CH), 86.3 (C), 58.5 (CH), 44.8 (CH_2), 41.1 (CH_2), 36.4 (CH_2), 19.3 (CH_2).

Cyclo-(Pro-Phe) (**6**): ^{1}H NMR (500 MHz, DMSO-d_6) δ 7.99 (1H, s), 7.23-7.30 (4H, m), 7.19 (1H, m), 4.35 (1H, t, J = 5.2 Hz), 4.06 (1H, m), 3.40 (3H, d, J = 9.8 Hz), 3.26 (1H, m), 3.06 (2H, m), 2.01 (1H, m), 1.71 (2H, m), 1.42 (1H, m); ^{13}C NMR (125 MHz, DMSO-d_6) δ 169.1 (C=O), 165.1 (C=O), 137.3 (C), 129.8 (CH), 128.0 (CH), 126.4 (CH), 58.4 (CH), 55.8 (CH), 44.6 (CH_2), 35.4 (CH_2), 27.8 (CH_2), 21.9 (CH_2).

(Z)-3-benzylidene-2-methylhexahydropyrrolo [1,2-1] pyrazine-1,4-dione (**7**): ^{1}H NMR (500 MHz, DMSO-d_6) δ 7.39 (2H, m), 7.33 (1H, m), 6.94 (1H, s), 4.43 (1H, m), 3.48 (1H, m), 2.72 (1H, s), 2.22 (1H, m), 1.98 (1H, m), 1.90 (1H, m); ^{13}C NMR (125 MHz, DMSO-d_6) δ 167.8 (C), 160.1 (C), 134.2 (C), 133.7 (C), 129.5 (CH), 128.4 (CH), 128.2 (CH), 118.7 (CH), 58.1 (CH), 44.9 (CH_2), 34.2 (CH_3), 27.5 (CH_2), 22.2 (CH_2).

Cyclo-(L-Ile-L-Pro) (**8**): ^{1}H NMR (500 MHz, DMSO-d_6) δ 7.95 (1H, s ,NH), 4.11 (1H, t, J = 7.5 Hz), 3.95 (1H, t-like), 3.40 (1H, m), 2.13 (1H, m, overlap), 2.02 (1H, m, overlap), 1.86 (1H, m, overlap), 1.83 (1H, m, overlap), 1.80 (1H, m, overlap), 1.34 (1H, m), 1.26 (1H, m), 0.98(3H, d, J = 7.2 Hz), 0.82(3H, t, J = 7.4 Hz); ^{13}C NMR (125 MHz, DMSO-d_6) δ 170.1 (C=O), 165.2 (C=O), 59.2 (CH), 58.2 (CH), 44.6 (CH_2), 34.9 (CH), 27.9 (CH_2), 23.9 (CH_2), 22.0 (CH_2), 15.0 (CH_3), 12.3 (CH_3).

Cyclo(D)-Pro-(D)-Leu (**9**): ^{1}H NMR (500 MHz, DMSO-d_6) δ 8.00 (1H, s, NH), 4.19 (1H, t, J = 8.0 Hz), 4.02 (1H, dd (7.15, 5.1), 3.37 (2H, m), 2.12(1H, m), 1.91 (1H, m), 1.85 (1H, overlap), 1.79 (2H, overlap), 1.74 (1H, overlap), 1.35 (1H, m), 0.87 (3H, d, J = 4.3 Hz), 0.86 (3H, d, J = 4.2 Hz); ^{13}C NMR (125 MHz, DMSO-d_6) δ 170.4 (C=O), 166.5 (C=O), 58.5 (CH), 52.6 (CH), 44.9 (CH_2), 37.8 (CH_2), 27.4 (CH_2), 24.1 (CH), 22.8 (CH_2), 22.5 (CH_3), 21.9 (CH_3).

Alterlactone (**12**): ^{1}H NMR (500 MHz, DMSO-d_6) δ 10.21 (1H, s), 9.46 (2H, s), 7.04 (1H, s), 6.92 (1H, s), 6.49 (2H, d, J = 24.6 Hz), 4.84 (2H, d, J = 15.9 Hz), 3.82 (3H, s); ^{13}C NMR (125 MHz, DMSO-d_6) δ 168.8 (C=O), 162.3 (C), 160.0 (C), 146.6 (C), 145.9 (C), 140.1 (C), 129.9 (C), 126.7 (C), 115.6 (CH), 109.5 (C), 105.1 (CH), 100.8 (CH), 67.8 (CH_2), 55.4 (OCH_3).

Penicillide (**13**): ^{1}H NMR (500 MHz, DMSO-d_6) δ 7.65 (1H, d, J = 8.5 Hz), 6.96 (1H, d, J = 8.5 Hz), 6.78 (1H, d, J = 2.1 Hz), 6.42 (1H, d, J = 2.1 Hz), 5.09 (2H, s), 4.91

(1H, m), 3.83 (3H, s) , 2.16 (2H, s) , 1.75 (1H, m) , 1.50 (1H, m) , 1.32 (1H, m) , 0.92 (3H, d, J = 6.6 Hz) , 0.88 (3H, d, J = 6.7 Hz); ^{13}C NMR (125 MHz, DMSO-d_6) δ 167.3 (C), 153.4 (C), 151.0 (C), 148.6 (C), 141.8 (C), 138.0 (C), 134.0 (C), 131.2 (CH), 127.0 (CH), 119.8 (CH), 119.0 (C), 118.3 (CH), 117.6 (CH), 68.7 (CH_2), 64.0 (CH) 61,9 (OCH_3), 48.0 (CH_2), 24.4 (CH) , 23.5 (CH_3) , 21.7 (CH_3) , 20.4 (CH_3).

Dehydroisopenicillide (**14**): ^{1}H NMR (500 MHz, DMSO-d_6) δ 7.75 (1H, d, J = 8.6 Hz), 6.94 (1H, d, J = 8.6 Hz), 6.77 (2H, m, overlap), 6.71 (1H, d, J = 10.7 Hz), 6.44 (1H, d, J = 16.1 Hz), 6.42 (1H, d, J = 3.4 Hz), 5.10 (2H, s), 4.78 (1H, s), 3.80 (3H, s) , 2.16 (3H, s) , 1.27 (6H, s); ^{13}C NMR (125 MHz, DMSO-d_6) δ 166.9 (C), 154.0 (C), 151.0 (C), 148.5 (C), 141.8 (C), 141.6 (C), 134.0 (C), 130.5 (C), 128.8 (C), 127.0 (C), 120.5 (C), 119.9 (CH), 119.0 (C), 118.3 (CH), 117.6 (CH), 69.4 (CH_2), 68.7 (CH_3), 62.1 (OCH_3), 30.0 (2CH_3), 20.4 (CH_3).

3′-*O*-Methyldehydroisopenicillide (**15**): ^{1}H NMR (500 MHz, DMSO-d_6) δ 7.81 (1H, d, J = 8.6 Hz), 6.95 (1H, d, J = 8.5 Hz), 6.78 (1H, d, J = 2.2 Hz), 6.65 (1H, d, J = 16.5 Hz), 6.43 (1H, d, J = 2.1 Hz), 5.11 (2H, s), 3.81 (4H, d, J = 5.2 Hz), 3.11 (3H, s), 2.16 (4H, s), 1.30 (6H, s); ^{13}C NMR (125 MHz, DMSO-d_6) δ 166.8 (C), 154.2 (C), 151.4 (C), 148.5 (C), 141.8 (C), 141.5 (CH), 138.0 (C), 134.0 (CH), 130.8 (C), 128.2 (C), 127.0 (CH), 121.1 (C), 120.5 (CH), 119.9 (CH), 118.3 (CH), 74.7 (C), 68.7 (CH_2), 62.2 (OCH_3), 49.8 (CH_3), 25.7 (CH_3), 20.4 (CH_3).

ITS sequence of *Talaromyces* sp. SCSIO 41431

ACTTTCTTCCCCCCTTTTGATATGCTTAAGTTCAGCGGGTAACTCCTACCTG
ATCCGAGGTCAACCGTGGTAAAATGTGGTGGTGACCAACCCCCGCAGGTC
CTTCCCGAGCGAGTGACAAAGCCCCATACGCTCGAGGACCAGACGGACGT
CGCCGCTGCCTTTCGGGCAGGTCCCCGGGGGGACCGCACCCAACACACAA
GCCGTGCTTGAGGGCAGAAATGACGCTCGGACAGGCATGCCCCCCGGAAT
GCCAGGGGGCGCAATGTGCGTTCAAAGATTCGATGATTCACGGAATTCTGC
AATTCACATTACTTATCGCATTTCGCTGCGTTCTTCATCGATGCCGGAACCA
AGAGATCCATTGTTGAAAGTTTTGACAATTTTCATACTACTCAGACAGCCCA
TCTTCATCAGGGTTCACAGAGCGCTTCGGCGGGCGCGGGCCCGGGGACGA

GCGTCCCCCGGCGACCAGGTGGCCCCGGTGGGCCCGCCAAAGCAACAGGT
GTATAGAGACAAGGGTGGGAGGTTGGGCCGCGAGGGCCCGCACTCGGTAA
TGATCCTTCCGAGTCC
